# The mediating role of anxiety and depression symptoms in the relationship between ADHD symptoms and polysubstance use among French university students: the i-share study

**DOI:** 10.1016/j.abrep.2025.100652

**Published:** 2025-12-13

**Authors:** François A.M. Jean, Charline Galesne, Noelia Retuerto, Marie C. Navarro, Zeineb Azouz, Agathe Tabyaoui, Mélissa Macalli, Christophe Tzourio, Cédric Galéra

**Affiliations:** a Dr Jean Eric Techer Hospital, Department of Child and Adolescent Psychiatry, Calais, France; b Lille Neuroscience et Cognition, UMR1172, BEEP Team, Platform Cure, National Institute of Health and Medical Research (Institut national de la santé et de la recherche médicale - INSERM), Lille, France; c University of Bordeaux, Bordeaux, France; d Bordeaux Population Health Research Center, UMR1219, HEALTHY Team, National Institute of Health and Medical Research (Institut national de la santé et de la recherche médicale - INSERM), Bordeaux, France; e Teaching Hospital of Bordeaux (Centre Hospitalier Universitaire de Bordeaux), Bordeaux, France; f Charles Perrens Hospital, Department of Child and Adolescent Psychiatry, Bordeaux, France

**Keywords:** Attention deficit hyperactivity disorder, Polysubstance use, Depression, Anxiety, Mediation

## Abstract

**Introduction:**

Polysubstance use, depressive disorder, and anxiety disorder are frequent comorbidities in adults with ADHD. This study aimed to explore the mediation role of anxiety and depression symptoms in the association between ADHD symptoms and polysubstance use.

**Methods:**

The study included 1675 French university students from February 2013 to July 2020 as part of the i-Share cohort. Time 1: ADHD symptoms were assessed using the Adult ADHD Self-Report Scale. Time 2: Anxiety and depression symptoms were evaluated using the State-Trait Anxiety Inventory-Y trait and the Patient Health Questionnaire-9, respectively. Time 3: Substance use was assessed through a self-reported questionnaire. We conducted a mediation analysis using structural equation modeling.

**Results:**

The study included 1675 participants. At time 1, the level of ADHD symptoms was moderate (m: 10.66, sd: 3.99). At time 2, both anxiety (m: 47.11, sd: 10.57) and depression (m: 6.92, sd: 5.33) symptoms were moderate. At time 3, 93.49% (1566) of participants had used substances. The direct effect between ADHD factor and polysubstance use factor was of 1.66 (1.04–2.48). The indirect effects between ADHD factor and polysubstance use factor were through anxiety factor −0.68 (−1.15 to −0.37) and through depression factor −0.57 (−0.94 to −0.32). Although the mediation effects through anxiety and depression factors were negative, the total effect remained positive and significant (0.41 (0.27 to 0.59)).

**Discussion:**

Anxiety factor and depression factor acted as negative and partial mediators in the association between ADHD factor and polysubstance use factor.

## Highlights

• ADHD factor is positively associated with polysubstance use factor.
• Anxiety and depression factors act as negative and partial mediators between ADHD factor and polysubstance use factor.
• Multigroup structural equation modeling revealed no significant differences based on sex.

1 Introduction

Attention deficit/hyperactivity disorder (ADHD) is a neurodevelopmental disorder and one of the most frequent mental disorders (GBD 2019 Mental Disorders Collaborators, 2022). Core symptoms of ADHD include hyperactivity, attention deficit and impulsivity (American Psychiatric Association, 2022). The prevalence of ADHD in adults is 2,5% (Simon et al., 2009).

ADHD is characterized by a high rate of psychiatric comorbidities which contribute significantly to the functional impairments associated with the condition. In the national comorbidity survey replication study conducted in the U.S.A., between 2001 and 2003, adults with ADHD had significantly higher rates of mood and anxiety disorders compared to those without ADHD. They were more likely to experience major depressive disorder (OR = 2.7, 95 % CI: 1.5–4.9), dysthymia (OR = 7.5, 95 % CI: 3.8–15.0), bipolar disorder (OR = 7.4, 95 % CI: 4.6–12.0), generalized anxiety disorder (OR = 3.2, 95 % CI: 1.5–6.9), post-traumatic stress disorder (OR = 3.9, 95 % CI: 2.1–7.3), panic disorder (OR = 3.0, 95 % CI: 1.6–5.9), and obsessive–compulsive disorder (OR = 1.5, 95 % CI: 0.2–9.4) (Kessler et al., 2006). Among these comorbidities, depressive and anxiety disorders were particularly prevalent.

Similarly, ADHD and substance use or substance use disorder constitute another key comorbidity associated with negative outcomes (Charach et al., 2011, Falck et al., 2006, Huntley and Young, 2014, Kaye et al., 2013, Lee et al., 2011, Miovský et al., 2021, Van Emmerik-Van Oortmerssen et al., 2012). Among adults with ADHD, the prevalence of any substance use disorder is 15.2 %, compared to 5.6 % in those without ADHD, with an OR of 3.7 (95 %CI: 2.2–6.2) (Kessler et al., 2006). The *meta*-analysis by Rohner et al. (2023) reported a combined ADHD prevalence of 21 % (95 % CI: 17–25 %) among adults with substance use disorder (Rohner et al., 2023). Additionally, Charach et al. found in their *meta*-analysis that childhood ADHD was prospectively associated with a higher risk of developing drug use disorder in adolescence or adulthood (combined OR: 3.48 (95 %CI 1.80–6.73)) (Charach et al., 2011). Patients with both ADHD and substance use disorder are more likely to discontinue treatment compared to those with substance use disorder alone, and addiction treatment is generally less effective for individuals with co-occurring ADHD (Daigre et al., 2021, Kolpe and Carlson, 2007, Levin et al., 2004, Patel et al., 2018).

Polysubstance use is particularly relevant when examining individuals engaged in substance use. Polysubstance use refers to the concurrent consumption of more than one substance over a defined period of time (Connor et al., 2014). Polysubstance use is not a disorder in itself and should be considered separately from substance use disorder. It is a common pattern of substance use and is associated with adverse outcomes. A *meta*-analysis, reported that, among cocaine users, the combined prevalence of simultaneous and co-occuring alcohol use was 74 % (95 % CI: 50–89) and 77 % (95 % CI: 62–87), respectively while corresponding rates for cannabis use were 38 % (95 % CI: 20–60) and 64 % (95 % CI: 47–79), respectively (Liu et al., 2018). The longitudinal Zurich Project on the Social Development from Childhood to Adulthood study reported that at age 20, 77 % of participants had experimented with more than one substance among alcohol, tobacco, or cannabis, while 36 % had experimented with more than one substance from a broader range of 14 substances, excluding alcohol and tobacco (Steinhoff et al., 2022). Compared to single substance use, polysubstance use is associated with higher risks of: depressive symptoms (Conway et al., 2013), health problems (Connor et al., 2014), deficits in cognitive functioning (Connor et al., 2014), smaller social networks (Kroll et al., 2018), traumatic injury (Compton et al., 2021), adverse driving events (Beaulieu et al., 2022), criminal justice involvement (Jones et al., 2023), and more severe patterns of substance use like addiction and overdose (Compton et al., 2021). Polysubstance use is therefore considered a marker of severity in substance use disorder. In addition, high levels of ADHD symptoms have been associated with multiple drug use (Capusan et al., 2019), and to a greater number of substances used (Jean et al., 2023). Liu et al. (Liu et al., 2018) emphasized the importance of studying polysubstance use stating: *“Limiting research to individual substances risks overlooking potentially critical interactions among substances, which may influence the patterns, consequences, and ultimately efficacy of treatment for substance use disorders.”(p. 16)*.

Several hypotheses may explain the prospective link between ADHD symptoms and polysubstance use. From a genetic perspective, ADHD and substance use share a background of common genetic variants (Gerring et al., 2024, Koller et al., 2024, Vilar-Ribó et al., 2021). From a neurobiological perspective, the dopaminergic pathway seems to be implicated in both ADHD and substance use, as suggested by findings that stimulants reduce substance abuse in persons with ADHD (Biederman, 2003, Faraone, 2018). From a cognitive perspective, a deficit in executive functions such as planning and response inhibition is particularly linked with ADHD (Schachar and Crosbie, 2024, Willcutt et al., 2005). Thus, impulsivity and deficits in executive functions may play a role in substance use (Jakubiec et al., 2022, Lozano-Madrid et al., 2020). From a social-environmental perspective, substance use is associated with parental separation and a lack of parental support during childhood (Jean et al., 2022). By extension, the ADHD symptoms–polysubstance use association could be the result of multiple additive and synergistic factors at different levels. As highlighted by the references above, ADHD is associated with depression and anxiety. ADHD could be a risk factor for depression and anxiety due to the accumulation of environmental stressors such as burnout (Turjeman-Levi et al., 2024), job-related problems (Fuermaier et al., 2021), marital conflict (Kahveci Oncu & Turatel Kislak, 2022), or involvement with the criminal justice system (Anns et al., 2023). In addition, depression and anxiety are common comorbidities of substance use disorder (Howard et al., 2019, Yoshimasu et al., 2016). Depression and anxiety symptoms could favor polysubstance use through self-treatment (Bolton et al., 2009, Robinson et al., 2009, Turner et al., 2018) or as strategies to cope with social inhibition and low self-esteem during social interaction (Dorard, Bungener, Corcos, & Berthoz, 2014). The question is whether, in persons with ADHD, associations between depression and anxiety symptoms and polysubstance use occur.

Mediation analysis provides a valuable framework for exploring the relationships between ADHD symptoms, depression symptoms, anxiety symptoms, and polysubstance use. However, findings in the literature have been inconsistent. A study on Chinese university students examined the separate mediating effects of anxiety and depression in the associations between hyperactivity and substance use, as well as between inattention and substance use. The study found that: anxiety and depression mediated the relationship between hyperactivity and both drinking and smoking behaviors. In contrast, only depression but not anxiety mediated the relationship between inattention and both drinking and smoking behaviors, but anxiety did not (Tong et al., 2016). Conversely, a longitudinal study found no significant mediation effect of depression on the association between childhood ADHD and adult substance use (Howard et al., 2019). Another longitudinal study found that adolescents with both depression and ADHD were not at significantly greater risk of developing substance use disorder than those with depression only (Yoshimasu et al., 2016).

Among the few studies available on the topic (Tong et al., 2016, Howard et al., 2019, Yoshimasu et al., 2016), only one study used latent variables, but it neither used bootstrapped confidence intervals nor considered attention deficit and hyperactivity simultaneously. Moreover, no studies have specifically explored polysubstance use in this context.

The objective of this study was to examine the mediating role of anxiety and depression in the association between ADHD and polysubstance use among French university students using factors to estimate conditions.

We hypothesized that psychiatric comorbidities, specifically anxiety and depression, would partially mediate the association between ADHD and polysubstance use.

2 Methods
  2.1 Study design

The present study is a prospective cohort study conducted as part of in the Internet-based Students’ Health Research Enterprise project (i-Share, https://www.i-share.fr). I-Share is a population-based study involving university students across France. It aims to explore academic life, health, substance use, family features, and the lifestyle of university students. Participants were recruited through promotional campaigns including social media, stands at registration events, university emails, flyers, and posters. They registered and completed the questionnaires online. There was an inclusion questionnaire (T1), a first follow-up questionnaire (T2) between 3 and 14 months after inclusion, and a second follow-up questionnaire (T3) between 24 and 42 months after inclusion. The i-Share project was approved by the French national regulatory agency (Commission nationale de l’informatique et des libertés, registration number [DR-2013-019]). The i-Share protocol was submitted to the regional ethics review board (Comité de protection des personnes, Sud-Ouest et Outre Mer III).

2.2 Population

Eligibility criteria were: being registered in French universities or higher education institutions, and being aged between 18 and 30 years. Inclusions took place between February 2013 and July 2020. All participants consented after receiving written information about the study. There were no financial rewards.

2.3 Data acquisition
  2.3.1 ADHD symptoms (T1)

At T1, participants completed the Adult ADHD Self-Report Scale French version 1.1 (ASRS) (Caci et al., 2008, Kessler et al., 2005). The ASRS explores ADHD symptoms. It is a short 6-items self-report questionnaire. We computed the total score, the hyperactivity subscore, and the attention deficit subscore. The ASRS has demonstrated internal validity, external validity, and reliability in English and other languages including French and in various populations such as university students (Caci et al., 2008, Caci et al., 2023, Gray et al., 2014, Green et al., 2018, Kessler et al., 2005, Kessler et al., 2007, Kiatrungrit et al., 2017, Van De Glind et al., 2013).

2.3.2 Anxiety symptoms (T2)

At T2, we assessed anxiety symptoms using the French version of the State Trait Anxiety Inventory − form Y trait part (STAI-Y2) (Spielberger et al., 1983, Spielberger et al., 1993). The trait part estimates the stress and worry that one experiences daily and stably. It does not estimate the reaction to a stress factor. It includes 20 self-report items. We computed a global score. The STAI-Y2 has been validated in different populations and different languages (Abdoli et al., 2020, Donham and Ludenia, 1984; C. Spielberger et al., 1983, Thomas and Cassady, 2021, Vitasari et al., 2011, Wiglusz et al., 2019).

2.3.3 Depression symptoms (T2)

At T2, we explored depression symptoms using the Patient Health Questionnaire − 9 (PHQ9) French version (*Patient Health Questionnaire (PHQ) Screeners*, n.d.). It a self-report questionnaire. It is based on the nine criteria for major depressive disorder of the Diagnostic and Statistical Manual of Mental Disorders – IVth edition criteria (American Psychiatric Association, 1998). A total score was calculated by summing the item responses. PHQ9 is reliable and has a good validity in French and other languages, and in different populations including university students (Adewuya et al., 2006, Carballeira et al., 2007, Kroenke et al., 2001).

2.3.4 Psychoactive substance uses (T1 and T3)

At T1 and T3, a series of questions were used to assess substance use during the preceding year. The assessed substances were: alcohol, tobacco, cannabis, cocaine, ecstasy, amphetamines, magic mushrooms, and other drugs. The following answers were available: “no”, “yes”, “I do not wish to reply.”. If the answer was “I do not wish to reply.”, the response was treated as a missing value. We created a variable called “Substance use” coded “no” if all psychoactive substance uses were coded “no”. Otherwise, this variable was coded “yes”. We also created a variable called “Number of substance use” summing the psychoactive substance uses with “no” transformed in 0 and “yes” transformed in 1.

2.4 Included participants and missing data management

A total of 17 520 participants completed the inclusion criteria at T1. At T2, 3789 participants completed the questionnaire, and at T3, 1687 participants remained. Among them, 1675 had complete data for all substance use questions at T3. Eleven variables comprised missing values. These missing values were missing at random. To address this, we performed multiple imputation using the MICE algorithm (Azur et al., 2011, van Buuren and Groothuis-Oudshoorn, 2011). All analyses were conducted on the imputed dataset.

2.5 Statistics
  2.5.1 Descriptive analysis

First, we described numerical variables using means (m) and standard deviations (sd), and categorical variables using percentages (%) and counts (n).

2.5.2 Main analysis: Structural equation modeling

Next, a preliminary analysis examined the individual effects of ASRS score at T1, PHQ9 score at T2, and STAI-Y2 at T2 on the number of substance use at T3, as well as the effect of ASRS score at T1 on PHQ9 at T2 and STAI-Y2 at T2.

Then, we performed structural equation modeling (SEM) to study the mediation effect of anxiety and depression symptoms in the association between ADHD symptoms and polysubstance use. SEM refers to *‘a set of equations with accompanying assumptions of the analyzed system, in which the parameters are determined on the basis of statistical* observation’*(p. 314)* (Tarka, 2018). Variables could be observed if they were measured or latent if they were constructed.

To study the mediating effect of anxiety and depression on the association between ADHD symptoms and polysubstance use within a SEM framework, we defined four latent variables: ADHD factor, polysubstance use factor, anxiety factor, and depression factor. The polysubstance use factor should be understood as inheriting the timeframe of the substance-use questions (the year preceding T3). Likewise, the depression and anxiety factors inherit the timeframe of the point-in-time assessment at T2. We used the regressions between these latent variables to estimate direct effects, indirect effects, and total effects. To select adjustment variables, we used a model with multivariate outcomes and multivariable predictors. ASRS total score at T1 and the square root number of substance use at T3 were defined as outcomes and the potential adjustment variables were the predictors. Age at T1, sex, and number of substance use at T1 were forced in the model and we considered all variables significantly associated with either ASRS total score at T1 or the number of substances used at T3. We applied a bidirectional stepwise selection keeping variables associated significantly with the both outcomes. We took into account the collinearity using a least absolute shrinkage and selection operator (LASSO) version of the model to remove collinear adjusting variables.

In SEM, latent variables are also referred as factors. Variables that predict others are called exogenous variables, and variables that are predicted are called endogenous variables. Observed variables predicted by factors are referred as indicators, and the coefficients of these relationships are called factor loadings. We followed several steps to perform SEM: specification of the model, identification of the model, fit evaluation of the model, modification of the model, determining the final model, and reporting of the results. Estimates are presented both raw with their confidence intervals and standardized. Robust standard errors for the estimations were computed using bootstrapping with 10,000 simulations. Model adequacy to the data was tested using a conventional chi-square test and various goodness-of-fit indices: standardized root mean squared residual (SRMR), root mean square error of approximation (RMSEA), confirmatory fit index (CFI), and Tucker-Lewis index (TLI) (Gana and Broc, 2019, Kyndt and Onghena, 2014).

2.5.3 Additional analysis: Multiple groups structural equation modeling

Finally, we examined the stability of the results in function of the sex. We used a multiple groups SEM (Jöreskog, 1971, Sörbom, 1974) with sex as the grouping variable and applied the same structure as the final SEM. This allowed us to study whether mediation effects differed between male and female. First we imposed a similar structure to the two groups (configural invariance) and we tested model adequacy. Then, we used a likelihood ratio test, the difference between the RMSEA (delta-RMSEA), and the difference between the TLI (delta-TLI) to assess various levels of invariance: weak, strong, strict, and structural invariances.

2.5.4 General informations on statistics

The first species risk was 5 %. Confidence intervals were at 95 %. Statistical tests were two-sided. Estimations were rounded at two digits except p-values which were rounded at three digits. We used R version 4.3.0 (R Core Team, 2023) for basic statistics and the lavaan package version 0.6–18 (Rosseel, 2012) for SEM. Draw.io was used for the SEM representations.

More information on data acquisition, covariables, missing data management, SEM, adjustment, and other statistical methods are detailed in supplementary material.

3 Results
  3.1 Sample description

Among the 1675 participants, 80.3 % (n: 1345) were female and the mean age was 20.27 years (sd: 2.15) at T1. The level of ADHD symptoms at T1 measured by the ASRS score, was moderate (m: 10.66, sd: 3.99). A total of 97.07 % (n: 1626) participants had used substances at T1 and 93.49 % (n: 1566) at T3. At T2, the levels of anxiety and depression symptoms were moderate (respectively, m: 47.11, sd: 10.57; and m: 6.92, sd: 5.33). More descriptive results are detailed in Table 1.

3.2 Determining the structural equation model
3.2.1 Individual effects

**Table 1:** Characteristics of the sample.

**Total sample (n = 1675)**	**% (n) or mean (sd)**

***Student variables at T1:***	
Sex: female	80.3 (1345)
Age (years)	20.27 (2.15)
Suicide attempt history	7.16 (120)
Depressive disorder history	9.79 (164)
Anxiety disorder history	13.13 (220)
***Scales:***	
ASRS at T1	10.66 (3.99)
ASRS attention deficit at T1	6.48 (3.16)
ASRS hyperactivity at T1	4.19 (1.91)
PHQ9 at T2	6.92 (5.33)
STAI-Y2 at T2	47.11 (10.57)
***Substance uses at T3:***	
Substance use	93.49 (1566)
Number of substance use	1.52 (1.04)
Alcohol use	92.96 (1557)
Tobacco use	16.12 (270)
Cannabis use	26.39 (442)
Cocaine use	3.82 (64)
Ecstasy use	4.96 (83)
Amphetamines use	0.72 (12)
Magic mushrooms use	2.03 (34)
Other drugs use	4.84 (81)

n: count; %: percentage; m: mean; sd: standard deviation; T1: inclusion; T2: first follow-up; T3: second follow-up; ASRS: Adult ADHD Self-Report Scale; PHQ9: Patient Health Questionnaire − 9; STAI-Y2: State Trait Anxiety Inventory − form Y trait part.

The ASRS score at T1 was significantly and positively associated with the PHQ9 score at T2 (estimate: 0.45, p < 0.001), the STAI-Y2 score at T2 (estimate: 0.93, p < 0.001), and the number of substance used at T3 (estimate: 0.02, p < 0.001). However, neither the PHQ9 score at T2 nor STAI-Y2 score at T2 was associated with the number of substances used at T3, with effects being quasi-null (respectively: estimate 0.00 and p: 0.976; estimate 0.00 and p: 0.487).

3.2.2 Specification

We defined four factors: ADHD, polysubstance use, anxiety, and depression. We assessed the different indicators for factors prospectively. Since ADHD symptoms are measured at T1, we considered it as the origin of the path. Anxiety and depression symptoms were assessed at T2. Substance uses were recorded at T3. Thus, the anxiety and depression factors were considered intermediates between ADHD factor and the polysubstance use factor. Polysubstance use factor was the final response. The ADHD factor was exogenous variable for polysubstance use factor, anxiety factor, and depression factor. Anxiety factor and depression factor were exogenous variables for the polysubstance use factor. The hyperactivity subscore and attention deficit subscore of ASRS were indicators for the ADHD factor and were treated as numerical variables. All substance uses were considered as indicators for the polysubstance use factor and were treated as ordinal variables. We scaled the polysubstance use factor on cocaine use because of the role played by cocaine use in polysubstance use (Liu et al., 2018). STAI-Y2 total score was considered as an indicator for the anxiety factor and treated as a numerical variable. PHQ9 total score was considered as an indicator for the depression factor and treated as a numerical variable. Intercepts for indicators and factors were introduced in the model. Adjusting variables were predictors for both ADHD factor and polysubstance use factor. There was no covariance between the adjusting variables.

3.2.3 Identification

There were 153 known values, 51 free parameters, 1 fixed parameter, and 103 degrees of freedom. The model was therefore over-identified and had no identification issues.

3.2.4 Evaluation

The goodness-of-fit test was significant (p: < 0.001) but not contributory due to the large sample size. The goodness-of-fit indices showed excellent model adequacy to the data for RMSEA (0.04 (CI95%: 0.04–0.04)), CFI (0.95), and TLI (0.94). However, SRMSR was too high (0.1). All coefficients are presented in Table S3 in the supplementary material.

3.2.5 Modification

To address the insufficient fit of SRMR, which indicated excess residual covariance, we added covariances between the different substance uses. In addition, we retained only the adjustment variables with at least one standardized coefficient greater than 0.3 or lesser than −0.3.

3.2.6 Identification and evaluation of the corrected model

With 43 degrees of freedom, the final model was over-identified. The goodness-of-fit test was significant (p: < 0.001), but this was not considered meaningful due to the large sample size. The goodness-of-fit indices showed excellent model adequacy, indicating good validity (SRMSR: 0.05, RMSEA: 0.03 (CI95%: 0.03–0.04), CFI: 0.98, TLI: 0.97).

3.2.7 Reporting factor construction

**Fig. 1:**
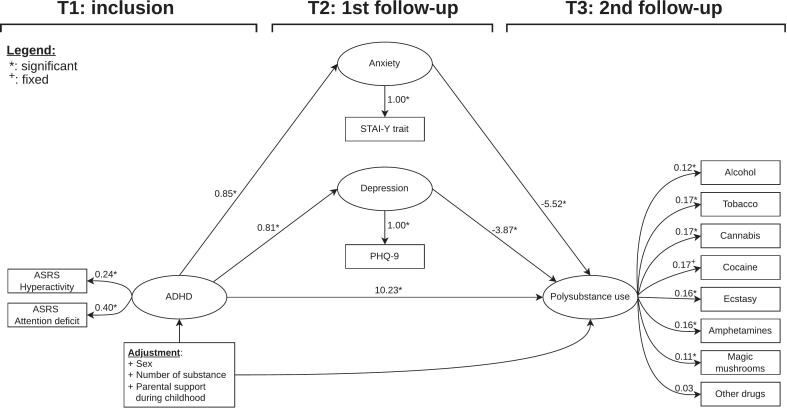
**Structural equation model integrating the association between ADHD and polysubstance use factors with a mediation by anxiety and depressive disorders factor.** The figure depicts the structural equation model. Ellipses represent factors, rectangles represent observed variables, and single arrows indicate regressions. Stars denote statistical significance based on bootstrapped confidence intervals. The estimations were standardized. Factor loadings for the ADHD and polysubstance use factors were significant, except for other drugs, even though their values varied between 0.11 and 0.4. There was a strong, positive, and statistically significant direct effect of the ADHD factor on the polysubstance use factor. Additionally, there was a positive and statistically significant association between the ADHD factor and the anxiety and depression factors. However, the anxiety and depression factors had a negative and statistically significant effect on the polysubstance use factor.

Fig. 1 illustrates the final model. The following raw loadings were oberved for: 1/ ADHD factor (ASRS attention deficit: 0.38 (0.28–0.47), hyperactivity: 0.38 (0.28–0.47)), 2/ anxiety factor (STAI-Y2: 3.39 (3.11–3.6)), 3/ depression factor (PHQ9: 2.35 (2.17–2.5)), and 4/ polysubstance use factor (alcohol: 0.54 (0.28–0.69), tobacco: 0.8 (0.7–0.91), cannabis: 0.91 (0.81–1.01), ecstasy: 1.05 (0.96–1.13), amphetamines: 1.11 (0.99–1.26), magic mushrooms: 0.73 (0.53–0.94), and other drugs: 0.33 (0.17–0.48)). ADHD factor had positive and statistically significant effects on the polysubstance use factor (raw: 1.66 (1.04–2.48), standardized: 2.41*), anxiety factor (raw: 2.08 (1.86–2.31), standardized: 0.83), and the depression factor (raw: 1.49 (1.33–1.65), standardized: 0.82). Anxiety and depression factors had negative and statistically significant effects on the polysubstance use factor (respectively: raw: −0.33 (−0.52 to −0.19), standardized: −1.17; and raw: −0.39 (−0.6 to −0.22), standardized: −1.01).

Further details on SEM specifications, estimations of the factors loadings, intercepts, slopes, thresholds, error variances, and covariances are presented in the supplementary material.

3.3 Mediation analysis

Using the SEM model, we performed a mediation analysis of depression and anxiety factors in the association between the ADHD factor and the polysubstance use factor. As reported in Table 2, the raw indirect effect of the ADHD factor on the polysubstance use factor through anxiety and depression factors were of −0.68 (−1.15 to −0.37) and −0.57 (−0.94 to −0.32) (standardized: −0.98 and −0.83). The direct effect of the ADHD factor on the polysubstance use factor was of 1.66 (1.04–2.48) (standardized: 2.41). Even though the mediating effects through the anxiety and depression factors were negative, the total effect is positive and statistically significant (raw: 0.41 (0.27–0.59), standardized: 0.6).

3.4 Additional analysis: Multiple groups SEM grouped by sex
  3.4.1 Specification, identification, and evaluation

**Table 2:** Association of ADHD and polysubstance use factors with mediation through Anxiety and Depression factors.

	**Raw Coefficient (95 %CI)**	**Standardized Coefficient**
***Final SEM:***		
Direct effect (ADHD −> Polysubtance use)	1.68 (1–2.57)	10.23
Indirect effect − Anxiety (ADHD −> Anxiety −> Polysubtance use)	−0.77 (−1.34 to −0.41)	−4.71
Indirect effect − Depression (ADHD −> Depression −> Polysubtance use)	−0.52 (−0.84 to −0.28)	−3.13
Total effect (ADHD => Polysubtance use)	0.39 (0.24–0.57)	2.39
***Multiple groups SEM:***		
***Female group***		
Direct effect (ADHD −> Polysubtance use)	1.02 (0.44–1.68)	1.82
Indirect effect − Anxiety (ADHD −> Anxiety −> Polysubtance use)	−0.39 (−0.7 to −0.16)	−0.69
Indirect effect − Depression (ADHD −> Depression −> Polysubtance use)	−0.37 (−0.69 to −0.11)	−0.65
Total effect (ADHD => Polysubtance use)	0.27 (0.13–0.42)	0.48
***Male group***		
Direct effect (ADHD −> Polysubtance use)	1.96 (0.68–8.76)	6.28
Indirect effect − Anxiety (ADHD −> Anxiety −> Polysubtance use)	−1.19 (−7.41 to −0.27)	−3.81
Indirect effect − Depression (ADHD −> Depression −> Polysubtance use)	−0.47 (−1.09 to −0.16)	−1.49
Total effect (ADHD => Polysubtance use)	0.3 (0.08–0.64)	0.97

CI: 10,000 iterations bootstrapped confident interval; =>: direct + indirect.

We performed the model using the same system as in the main analysis. We applied configural invariance, imposing the same factor structure on both groups. There were 420 known values, 150 free parameters, 2 fixed parameters, and 272 degrees of freedom. The model was therefore over-identified and had no identification issues. The goodness-of-fit indices showed excellent model adequacy (SRMSR: 0.05, RMSEA: 0.02 (CI95%: 0.01–0.03), CFI: 0.99, TLI: 0.99).

3.4.2 Invariance testing

**Fig. 2:**
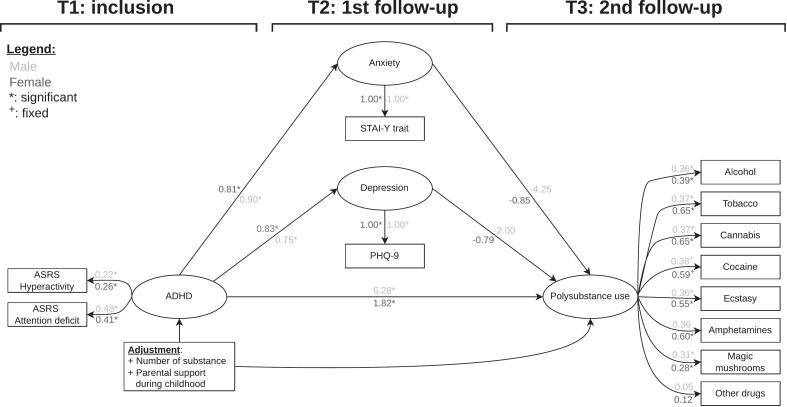
**Multiple groups structural equation model of the association between ADHD and polysubstance use factors with a mediation by anxiety and depressive disorders factor grouped by sex.** The figure depicts the structural equation model. Ellipses represent factors, rectangles represent observed variables, and single arrows indicate regressions. Stars denote statistical significance based on bootstrapped confidence intervals. The estimations are standardized. The coefficients for males and females were similar, and except for the factor loading on amphetamines, there was no change in significance.

The goodness-of-fit for the configural model was excellent showing a configural invariance between the groups. The test between the configural invariance model and the weak invariance model was not significant (p 0.396, delta-RMSEA: 0, delta-TLI: 0). Other tests for weak, strong, strict, and structural invariance were impossible due to the models’ failure to converge. The absence of evidence for non-invariance suggests no difference between females and males in the tested parameters. Therefor, groups should not be analyzed separately. Fig. 2 displays the multiple groups SEM and Table 2 presents the mediation analysis for males and females.

3.4.3 Comparison between females and males

Fig. 2 displays the multiple groups SEM. As reported in Table 2, the indirect effects between ADHD factor and polysubstance use factor were higher for males (depression path: raw: −0.47 (−1.09 to −0.16), standardized: −1.49; anxiety path: raw: −1.19 (−7.41 to −0.27), standardized: −3.81) than for females (depression path: raw: −0.37 (−0.69 to −0.11), standardized: −0.65; anxiety path: raw: −0.39 (−0.7 to −0.16), standardized: −0.69), but significant for females and for males. The direct effect between ADHD factor and polysubstance use factor was higher in males (raw: 1.96 (0.68–8.76), standardized: 6.28) than in females (raw: 1.02 (0.44–1.68), standardized: 1.82), and significant for both groups.

4 Discussion
  4.1 Main findings

This study aimed to investigate the mediating role of anxiety and depression symptoms in the relationship between ADHD symptoms and polysubstance use. The direct effect of the ADHD factor on the polysubstance use factor was positive and significant, whereas the indirect effects through anxiety and depression factors were negative and significant, indicating a negative mediating effect. Grouped analysis by sex showed no evidence sex dependence.

4.2 Interpretation and comparison with previous studies

The direct effect of the ADHD factor on the polysubstance use factor remained positive and significant, despite the negative mediating effects of anxiety and depression factors. This suggests that ADHD symptoms may play a primary role in the development of polysubstance use, potentially tipping the balance towards polysubstance use in people already vulnerable due to other risk factors (Connor et al., 2014). The impulsivity and lack of inhibition associated with ADHD (American Psychiatric Association, 2022) may underlie this primary role. Supporting this, a functional magnetic resonance imaging study found that the interaction between ADHD and cannabis use was linked to the activation in the right hippocampus and cerebellar vermis during correct response inhibition (Rasmussen et al., 2016). Additionally, a study including 101 participants found that individuals with ADHD and cocaine dependence exhibited poorer executive function performance compared to participants with cocaine dependence only, who in turn exhibited poorer performance than participants without any disorder (Cunha et al., 2013; Crunelle et al., 2013).

The negative indirect effects of the anxiety and depression factors in the ADHD-polysubstance use association were unexpected. Indeed, depressive and anxiety disorders are positively and prospectively associated with substance use disorders (Howard et al., 2019, Yoshimasu et al., 2016). In the indirect effects, the first associations between the ADHD factor and the anxiety or depression factors were positive and significant, but the second associations between the anxiety or depression factors and the polysubstance use factor were negative and significant. How can these negative and significant associations be understood? When considered separately, there were quasi-null and non-significant associations between PHQ-9 score or STAI-Y2 score and the number of substances used. However, these associations became negative and significant after including ADHD in the model suggesting a reversal paradox, where the association between two variables is reversed after adjusting for a third variable (Tu et al., 2008). Thus, the presence of the ADHD factor in the model changed the direction of the association between the depression or anxiety factors and the polysubstance use factor. The underlying mechanism of this effect is uncertain. In individuals with ADHD, the depression-polysubstance use association or the anxiety-polysubstance use association may differ from those observed in individuals without ADHD. Supporting this evidence, previous studies found no significant mediating effect of depression on the association between childhood ADHD and adult substance use or substance use disorder (Howard et al., 2019, Yoshimasu et al., 2016). These results, although showing null rather than negative effects, suggest the specificity in comorbidity patterns of ADHD individuals.

One possible explanation is that depression and anxiety may inhibit certain features of ADHD that normally facilitates polysubstance use, such as impulsivity and sensation-seeking behaviors. Several theoretical frameworks may help explain the suppression effect observed. The self-medication hypothesis suggests that anxiety and depressive symptoms can promote substance use as a maladaptive coping strategy (Bolton et al., 2009, Robinson et al., 2009, Turner et al., 2018). Conversely, internalising symptoms are also associated with social withdrawal and behavioural inhibition, which may reduce opportunities for substance use (Dorard, Bungener, Corcos, & Berthoz, 2014). ADHD, by contrast, is linked to impulsivity and sensation-seeking, which strongly increase the risk of polysubstance use (Jakubiec et al., 2022, Lozano-Madrid et al., 2020). In a predominantly female sample, where internalising symptoms are more prevalent, these opposing pathways may coexist, to the extent that controlling for ADHD reveals a negative indirect effect of internalising symptoms on polysubstance use.

5 Strengths and limitations
  This study has several key strengths. First, the sample size is large, which allows for more accurate estimations, reduced margins of error, and increased the ability to detect significant associations. Second, the use of SEM for mediation analysis provides a robust statistical framework for investigating mediating effects (Brown & Little, 2015). Third, we collected data on a wide range of substance use strengthening the validity of the polysubstance use factor. Fourth, the prospective measurement of variables made causal inferences more reliable and enhanced the robustness of mediation analysis.
  This study also has several limitations. First, the study sample consisted of university students in France, which may not be representative of all young adults. The sample included a very large majority of women, which is atypical for an ADHD study. These particularities may introduce selection bias and limit the generalizability of the findings to the broader young adult population. Second, although the study used a prospective design in adulthood, it did not account for the cumulative impact of ADHD during childhood and adolescence on polysubstance substance use. Third, the association between polysubstance use factor and anxiety and depression factors could be bidirectional. To mitigate this potential confounding effect, we adjusted the relationship between the ADHD factor and the polysubstance use factor for the number of substances used at T1. Fourth, the SEM approach relies on the adequacy of model specification with the data and does not account for external validity. Replicating this SEM model in different populations could enhance the external validity of the model specification used in this study. Fifth, ADHD, anxiety, and depression were not assessed through formal diagnostic interviews, but rather through self-reported symptom scales administered online. Although these instruments have demonstrated good validity in relation to the respective disorders (Adewuya et al., 2006, Carballeira et al., 2007, Kroenke et al., 2001, Abdoli et al., 2020, Donham and Ludenia, 1984; C. Spielberger et al., 1983, Thomas and Cassady, 2021, Vitasari et al., 2011, Wiglusz et al., 2019, Caci et al., 2008, Caci et al., 2023, Gray et al., 2014, Green et al., 2018, Kessler et al., 2005, Kessler et al., 2007, Kiatrungrit et al., 2017, Van De Glind et al., 2013), our findings should be interpreted as reflecting symptom severity rather than clinician-confirmed diagnoses and SEM was used subsequently to construct the ADHD, depression, anxiety, and polysubstance use factors. ADHD, depression, and anxiety factors are not formal diagnoses. Sixth the polysubstance use factor was from constructed binary responses (yes/no) to questions on the use of psychoactive substances. Although the SEM model demonstrated good fit with the data and supported the existence of the polysubstance use factor, the polysubstance use factor inherits the limitations of its indicators. It contains no information on frequency or quantity of use, and therefore cannot be linked to level of use. Furthermore, it is not informative as to addiction symptoms and hence cannot be used to assess substance use disorder.
5.1 Implications and unanswered questions
  Our findings have clinical and public health implications. Since ADHD factor plays a more significant role than anxiety and depression factors in polysubstance use factor, early screening, diagnosis, and treatment of ADHD in young adults, especially university students, should be reinforced to prevent substance use disorders. This is of utmost importance given that ADHD in adults is often underdiagnosed, especially among those seeking treatment for substance use (Crunelle et al., 2018; Ginsberg et al., 2014, Huntley et al., 2012). It appears highly relevant to train general practitioners, psychiatrists, and addiction specialists to better identify ADHD in young adults with substance use problems. In addition, it seems important to develop university-based support programs for students with ADHD, including mental health literacy, stress management tools, and substance use prevention workshops.
  Given that our study focused on young adults, future research could investigate whether the mediation effect of anxiety and depression in the association between ADHD and polysubstance use persists throughout the lifespan and across different age groups including children, adolescents, middle-aged adults, and older adults. In addition, future studies could further disentangle these associations by examining alcohol, tobacco, and illicit drug use separately.
6 Conclusion
  The negative mediating effect of anxiety and depression factors in the ADHD factor-polysubstance use factor association suggests that these depression and anxiety symptoms could slightly attenuate the influence of ADHD symptoms on polysubstance use. The findings that ADHD factor itself plays a more prominent role than comorbidities such as anxiety and depression factors in the co-occurrence of ADHD factor and polysubstance factor use among young adults underscores the need to enhance clinical and public health interventions tailored to this population.

**Ethics approval statement**

The i-Share project was approved by the French national regulatory agency (Commission nationale de l’informatique et des libertés, CNIL, registration number [DR-2013-019]). The i-Share protocol was submitted to the regional ethics review board (Comité de protection des personnes, Sud-Ouest et Outre Mer III, CPP).

**Participant consent statement**

All participants signed an online informed consent.

**Permission to reproduce material from other sources**

Not applicable.

**AI use statement**

We used ChatGPT (https://chatgpt.com/) to correct English language errors by adding the prompt ‘Is English correct:’ followed by the text.

**Registration**

This study was not preregistered.

Funding statement

The i-Share project was funded by the program ‘Investments for the Future’ (reference ANR-10-COHO-05). The i-Share Project is currently supported by an unrestricted grant from the Nouvelle-Aquitaine Regional Council (Conseil Régional Nouvelle-Aquitaine, grant N° 4370420) and by the Bordeaux ‘Initiatives d’excellence’ (IdEx) program of the University of Bordeaux (ANR-10-IDEX-03–02). It has also received grants from the Nouvelle-Aquitaine Regional Health Agency (Agence Régionale de Santé Nouvelle-Aquitaine, grant N°6066R-8), Public Health France (Santé Publique France, grant N°19DPPP023-0), The National Institute against cancer INCa (grant N°INCa_11502), and the Medical Research Foundation (Fondation pour la recherche). The funding bodies were involved neither in the study design nor in the collection, analysis, or interpretation of the data.

## CRediT authorship contribution statement

**François A.M. Jean:** Writing – review & editing, Writing – original draft, Visualization, Validation, Software, Methodology, Formal analysis, Data curation, Conceptualization. **Charline Galesne:** Writing – review & editing, Validation, Software, Formal analysis. **Noelia Retuerto:** Writing – review & editing, Validation, Software, Formal analysis. **Marie C. Navarro:** Writing – review & editing, Validation. **Zeineb Azouz:** Writing – review & editing, Validation. **Agathe Tabyaoui:** Writing – review & editing, Validation. **Mélissa Macalli:** Writing – review & editing, Visualization. **Christophe Tzourio:** Writing – review & editing, Validation, Resources, Project administration, Methodology, Funding acquisition. **Cédric Galéra:** Writing – review & editing, Writing – original draft, Validation, Supervision, Resources, Methodology, Formal analysis, Conceptualization.

## Declaration of competing interest

The authors declare that they have no known competing financial interests or personal relationships that could have appeared to influence the work reported in this paper.

## Data Availability

Data will be made available on request.
