# Supplementary material for: The mediating role of anxiety and depression symptoms in the relationship between ADHD symptoms and polysubstance use among French university students: the i-share study
Source: Addict Behav Rep. 2025 Dec 13;23:100652. doi: 10.1016/j.abrep.2025.100652 (PMC12767792; doi:10.1016/j.abrep.2025.100652)
Supplement: Supplementary Data 1 [file mmc1.docx]

Supplementary material

Table of contents

[1. Supplementary methods 3](#__RefHeading___Toc6172_4167346754)

[1.1 Data acquisition 3](#__RefHeading___Toc6174_4167346754)

[ADHD symptoms 3](#__RefHeading___Toc6176_4167346754)

[Anxiety symptoms (T2) 3](#__RefHeading___Toc6178_4167346754)

[Depression symptoms (T2) 4](#__RefHeading___Toc6180_4167346754)

[Psychoactive substance uses (T1 and T3) 4](#__RefHeading___Toc6182_4167346754)

[Covariables 5](#__RefHeading___Toc6184_4167346754)

[1.2 Missing data management 6](#__RefHeading___Toc6186_4167346754)

[Figure S1: Flowchart of the participants 7](#__RefHeading___Toc6188_4167346754)

[Table S1: Description of the available data, comparison of complete cases and incomplete cases, and univariate comparison of imputed data with descriptive statistics of available data 9](#__RefHeading___Toc6190_4167346754)

[1.3 Individual effects 13](#__RefHeading___Toc6192_4167346754)

[1.4 Structural equation modeling, detailed method 14](#__RefHeading___Toc6194_4167346754)

[1.5 Multiple groups structural equation modeling, detailed method 16](#__RefHeading___Toc6196_4167346754)

[1.6 General informations on statistics 16](#__RefHeading___Toc6198_4167346754)

[2. Supplementary results 17](#__RefHeading___Toc6200_4167346754)

[2.1 Description of the sample 17](#__RefHeading___Toc6202_4167346754)

[Table S2: Full description of the sample (n = 1675) 17](#__RefHeading___Toc6204_4167346754)

[2.2 Individual effects 20](#__RefHeading___Toc6206_4167346754)

[Table S3: Effects of the mediation analysis in univariate univariable regression models 20](#__RefHeading___Toc6208_4167346754)

[2.3 Multinormality 21](#__RefHeading___Toc6210_4167346754)

[2.4 Structural equation modeling, first model 22](#__RefHeading___Toc6212_4167346754)

[Figure S2: First SEM 22](#__RefHeading___Toc6214_4167346754)

[Table S4: Detailed parameters of the first SEM 22](#__RefHeading___Toc6216_4167346754)

[2.5 Structural equation modeling, final model 27](#__RefHeading___Toc6218_4167346754)

[Table S5: Detailed parameters of the final SEM 27](#__RefHeading___Toc6220_4167346754)

[2.6 Structural equation modeling, multiple groups 32](#__RefHeading___Toc6222_4167346754)

[Table S6: Detailed parameters of multiple groups structured equation modeling 32](#__RefHeading___Toc6224_4167346754)

[References 38](#__RefHeading___Toc6226_4167346754)

**Table count: 5**
**Figure count: 2**

# 1. Supplementary methods

## 1.1 Data acquisition

### ADHD symptoms

At T1, participants completed the Adult ADHD Self-Report Scale 1.1 (ASRS), French version (Caci, Bayle, & Bouchez, 2008; Kessler et al., 2005). The ASRS explores ADHD symptoms. It is a short 6-items self-report questionnaire based on the Diagnostic and Statistical Manual of Mental Disorders – IVth edition criteria (American Psychiatric Association, 1998). The scale explores six symptoms: 1/How often do you have trouble wrapping up the final details of a project, once the challenging parts have been done?; 2/How often do you have difficulty getting things in order when you have to do a task that requires organization?; 3/How often do you have problems remembering appointments or obligations?; 4/When you have a task that requires a lot of thought, how often do you avoid or delay getting started?; 5/How often do you fidget or squirm with your hands or feet when you have to sit down for a long time?; 6/How often do you feel overly active and compelled to do things, like you were driven by a motor?. Each question assesses how often the symptom of ADHD has occurred over the past 6 months using a 5-point Likert scale (0 = never; 1 = rarely; 2 = sometimes; 3 = often; 4 = very often). We computed the global score by adding the 6 items, the attention deficit subscore by adding the first 4 items, and the hyperactivity subscore by adding the last 2 items. High scores and subscores indicate a high level of ADHD symptoms.

The internal validity, external validity, and reliability of the ASRS have been demonstrated in English, French, and other languages, as well as in various populations such as university students (Caci et al., 2008; Caci, Didier, & Wynchank, 2023; Gray, Woltering, Mawjee, & Tannock, 2014; Green et al., 2018; Kessler et al., 2005; Kessler et al., 2007; Kiatrungrit, Putthisri, Hongsanguansri, Wisajan, & Jullagate, 2017; Van De Glind et al., 2013). The sensitivity and specificity of the ASRS were 68.7% and 97.9%, respectively (Kessler et al., 2005). The interrater reliability was substantial (Cohen’s kappa = 0.76) (Kessler et al., 2005; McHugh, 2012). The ASRS reliability was intermediate (Cronbach’s alpha = 0.63) (Kessler et al., 2007). The test-retest stability was moderate in the first study (Pearson’s correlation coefficient = 0.63) (Kessler et al., 2007) and high in a second study (Spearman’s rho = 0.78, intraclass correlation = 0.75) (Silverstein, Alperin, Faraone, Kessler, & Adler, 2018).

### Anxiety symptoms (T2)

At T2, we assessed anxiety symptoms using the French version of the State-Trait Anxiety Inventory, form Y, trait part (STAI-Y2) (C. D. Spielberger, Bruchon-Schweitzer, & Paulhan, DL 1993, cop. 1993; C. Spielberger, Gorsuch, Lushene, Vagg, & Jacobs, 1983). The trait part estimates the stress and worry that one experiences daily and stably. It does not estimate the reaction to a stress factor. It includes 20 self-report items. Participants were asked to think about how they generally feel when answering the items. Each item is scored on a 4-point Likert scale (1 = almost never, 2 = sometimes, 3 = often, 4 = almost always). We computed the total score by adding the answers for each item. Low scores indicate a mild form of anxiety, and high scores indicate a severe form of anxiety.

The STAI-Y2 has been validated in different populations and languages (Abdoli et al., 2020; Donham & Ludenia, 1984; C. Spielberger et al., 1983; Thomas & Cassady, 2021; Vitasari, Wahab, Herawan, Othman, & Sinnadurai, 2011; Wiglusz, Landowski, & Cubała, 2019). It demonstrated excellent internal consistency through confirmatory factor analysis in university students (Thomas & Cassady, 2021) and had a Cronbach’s alpha of 0.80 (Vitasari et al., 2011). The external validity was found to be 0.62 (correlation r) with the Beck Anxiety Inventory (Abdoli et al., 2020). The STAI-Y2 has also been validated in populations with alcohol use disorder (Donham & Ludenia, 1984).

### Depression symptoms (T2)

At T2, we explored depression symptoms with the Patient Health Questionnaire - 9 (PHQ9) French version (“Patient Health Questionnaire (PHQ) Screeners,” n.d.). It is based on the nine criteria for major depressive disorder of the Diagnostic and Statistical Manual of Mental Disorders – IVth edition criteria (American Psychiatric Association, 1998). The questionnaire asks, concerning the last 2 weeks, how often people have been bothered by any of the items. The French version includes nine self-report items with item nine in two parts. Each item is scored on a 4-point Likert scale (0 = not at all, 1 = several days, 2 = more than half of the days, 3 = nearly every day). We computed the total score by summing the first eight items and taking the maximum score from the two parts of item nine. The higher the score, the more severe the level of depression.

The PHQ9 is reliable and has good validity in English and other languages, and in different populations, including university students (Adewuya, Ola, & Afolabi, 2006; Carballeira et al., 2007; Kroenke, Spitzer, & Williams, 2001; Sun et al., 2020). The PHQ9 demonstrated good external validity with the 20-item Short-Form General Health Survey, the Hamilton Depression Scale (correlation r: 0.61), and the Beck Depression Inventory (correlation r: 0.67) (Adewuya et al., 2006; Kroenke et al., 2001; Sun et al., 2020). The internal consistency was high, with a Cronbach’s alpha of 0.85 in one study and 0.88 in another (Adewuya et al., 2006; Sun et al., 2020). The test-retest correlation was high (correlation r: 0.89 or 0.74) (Adewuya et al., 2006; Sun et al., 2020). A PHQ9 score greater than or equal to 10 had a sensitivity of 88% and a specificity of 88% (Kroenke et al., 2001). Finally, Doi et al. showed a bifactor structure and scalar invariance between different populations using confirmatory factor analysis (Doi, Ito, Takebayashi, Muramatsu, & Horikoshi, 2018).

### Psychoactive substance uses (T1 and T3)

At T1 and T3, a series of questions assessed the use of substances. The substances assessed were alcohol, tobacco, cannabis, cocaine, ecstasy, amphetamines, magic mushrooms, and other drugs. The following answers were available: “no,” “yes,” “I do not wish to reply.” If the answer was “I do not wish to reply,” the response was treated as a missing value. We created a variable called “Substance use,” coded as “no” if all psychoactive substance uses were coded as “no.” Otherwise, this variable was coded as “yes.” We also created a variable called “Number of substance uses” by summing the psychoactive substance use, with “no” transformed into 0 and “yes” transformed into 1.

### Covariables

We collected at T1: 1/ demographic characteristics: age (numerical), sex (male/female); 2/ student-related variables: academic level (first three years/fourth year or higher education), secondary education degree type (general/technical), deprived students’ social support (yes/no), job activity (yes/no); 3/ family-related variables: number of siblings (numerical), parental academic level (graduate or undergraduate studies/postgraduate studies), parental separation (yes/no), parental support during childhood (sparsely/a lot); 4/ comorbidities: reading disorder history (yes/no), disability history (yes/no); 5/ psychiatric history: suicidal attempt history (yes/no), depression history (yes/no), obsessive-compulsive disorder history (yes/no), anxiety disorder history (yes/no), eating disorder history (yes/no).

## 1.2 Missing data management

There were 17520 participants that completed the inclusion criteria at T1. At T2, 3789 participants completed the questionnaire and at T3, there were 1687. Among them, 1675 had no missing data for all substance use questions at T3. Figure S1 presents the flow chart of participants.

###
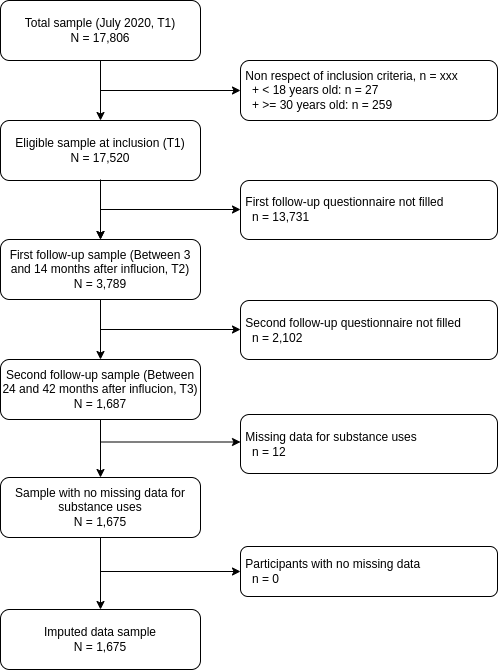
Figure S1: Flowchart of the participants

The figure depicts the flowchart of participants.

There were 11 variables that included missing values (cannabis use at T1: 40.1% (n= 682), cocaine use at T1: 0.6% (n= 10), ecstasy use at T1: 0.4% (n= 7), amphetamine use at T1: 0.5% (n= 8), magic mushroom use at T1: 7x2.2% (n= 1209), other drug use at T1: 72.2% (n= 1209), parental academic level: 0.8% (n= 14), parental separation: 2.5% (n= 41), parental support during childhood: 1.1% (n= 18), PHQ-9 total score at T2: 0.1% (n= 2), STAI-Y trait total score at T2: 0.1% (n= 1)). We tested whether variables with missing values were missing completely at random or missing at random. We compared complete cases and incomplete cases and tested the association between all variables and variables with missing values, binarized on missing values. We used the Wilcoxon rank-sum test with continuity correction for numerical variables and the Pearson Chi-squared test with Monte Carlo simulated p-values based on 2,000 replications for categorical variables. Variables were assumed to be missing at random.

We performed imputations with the MICE algorithm (Azur, Stuart, Frangakis, & Leaf, 2011; van Buuren & Groothuis-Oudshoorn, 2011) on 50 datasets with 50 iterations per dataset. We used the mice package (van Buuren & Groothuis-Oudshoorn, 2011). To check the imputed data, we performed a univariate comparison of the imputed data with descriptive statistics of the available data. We used a one-sample asymptotic mean test for numeric variables and an exact multinomial test for categorical variables. Missing values per variable, descriptions of available data, tests for the type of missing data, and tests for the quality of imputation are presented in Table S1.

### Table S1: Description of the available data, comparison of complete cases and incomplete cases, and univariate comparison of imputed data with descriptive statistics of available data

|  | **N missing** | **N available** | **% (n) or m (sd)** | **Comparison of lines with missing data and lines without missing data: p** | **Univariate comparison of imputed data with descriptive statistics of available data: p** |
| --- | --- | --- | --- | --- | --- |
| ***Student variables at T1:*** |  |  |  |  |  |
| Sex: female | 0 | 1675 | 80.3 (1345) | 0.004 | 1 |
| Age (years) | 0 | 1675 | 20.27 (2.15) | 0.979 | 0.939 |
| Academic level: first three years | 0 | 1675 | 72.54 (1215) | 0.073 | 1 |
| Secondary education degree type: technical | 0 | 1675 | 95.76 (1604) | 0.204 | 1 |
| Deprived students' Social support | 0 | 1675 | 40.18 (673) | 0.523 | 1 |
| Job activity | 0 | 1675 | 39.58 (663) | 0.139 | 1 |
| ***Family variables at T1:*** |  |  |  |  |  |
| Number of siblings | 0 | 1675 | 1.67 (1.08) | 0.604 | 0.977 |
| Parental academic level: postgraduate | 14 | 1661 | 58.46 (971) | 0.864 | 0.96 |
| Parental separation | 41 | 1634 | 28.21 (461) | 0.352 | 0.957 |
| Parental support during childhood: a lot | 18 | 1657 | 76.4 (1266) | 0.589 | 0.84 |
| ***Psychiatric conditions at T1:*** |  |  |  |  |  |
| Suicide attempt history | 0 | 1675 | 7.16 (120) | 0.598 | 1 |
| Depression history | 0 | 1675 | 9.79 (164) | 0.267 | 1 |
| Anxiety disorder history | 0 | 1675 | 13.13 (220) | 1 | 1 |
| Eating disorder history | 0 | 1675 | 3.64 (61) | 0.292 | 1 |
| Obsessive compulsive disorder history | 0 | 1675 | 1.73 (29) | 0.526 | 0.925 |
| ***Scales:*** |  |  |  |  |  |
| ASRS at T1 | 0 | 1675 | 10.66 (3.99) | - | 0.968 |
| ASRS attention deficit at T1 | 0 | 1675 | 6.48 (3.16) | - | 0.969 |
| ASRS hyperactivity at T1 | 0 | 1675 | 4.19 (1.91) | - | 0.947 |
| PHQ9 at T2 | 2 | 1673 | 6.92 (5.33) | - | 0.982 |
| STAI-Y2 at T2 | 1 | 1674 | 47.1 (10.57) | - | 0.979 |
| ***Substance uses at T1:*** |  |  |  |  |  |
| Substance use | 0 | 1675 | 92.48 (1549) | - | < 0.001 |
| Number of substance use | 0 | 1675 | 1.54 (1.13) | - | < 0.001 |
| Alcohol use | 0 | 1675 | 92 (1541) | < 0.001 | 1 |
| Tobacco use | 0 | 1675 | 20.3 (340) | < 0.001 | 1 |
| Cannabis use | 672 | 1003 | 46.96 (471) | < 0.001 | < 0.001 |
| Cocaine use | 10 | 1665 | 3.18 (53) | < 0.001 | 0.781 |
| Ecstasy use | 7 | 1668 | 6 (100) | < 0.001 | 1 |
| Amphetamines use | 8 | 1667 | 1.56 (26) | 0.003 | 1 |
| Magic mushrooms use | 1209 | 466 | 9.23 (43) | 1 | < 0.001 |
| Other drugs use | 1209 | 466 | 2.79 (13) | 0.04 | < 0.001 |
| ***Substance uses at T3:*** |  |  |  |  |  |
| Substance use | 0 | 1675 | 93.49 (1566) | - | 1 |
| Number of substance use | 0 | 1675 | 1.52 (1.04) | - | 0.944 |
| Alcohol use | 0 | 1675 | 92.96 (1557) | 0.001 | 0.962 |
| Tobacco use | 0 | 1675 | 16.12 (270) | 0.002 | 1 |
| Cannabis use | 0 | 1675 | 26.39 (442) | 0.001 | 1 |
| Cocaine use | 0 | 1675 | 3.82 (64) | 0.024 | 1 |
| Ecstasy use | 0 | 1675 | 4.96 (83) | < 0.001 | 1 |
| Amphetamines use | 0 | 1675 | 0.72 (12) | 0.306 | 1 |
| Magic mushrooms use | 0 | 1675 | 2.03 (34) | 0.002 | 1 |
| Other drugs use | 0 | 1675 | 4.84 (81) | 0.706 | 1 |
| n: count; %: percentage; m: mean; sd: standard deviation; p: p-value; -: test not computable; T1: inclusion; T2: first follow-up; T3: second follow-up; ASRS: Adult ADHD Self-Report Scale; PHQ9: Patient Health Questionnaire - 9; STAI-Y2: State Trait Anxiety Inventory - form Y trait part p-values from Wilcoxon rank sum test with continuity correction, Pearson's Chi-squared test with simulated p-value, One-sample asymptotic mean test, and Exact binomial test | | | | | |

## 1.3 Individual effects

We tested the individual effects of ASRS score at T1, PHQ9 score at T2, and STAI-Y2 score at T2 on the number of substance use instances at T3 using a generalized linear model with a Poisson link. Additionally, we tested the effect of ASRS score at T1 on PHQ9 score at T2 and STAI-Y2 score at T2 using a linear model.

## 1.4 Structural equation modeling, detailed method

We performed a structural equation modeling (SEM) to study the mediation effect of anxiety and depressive ~~disorders~~ symptoms in the association between ADHD symptoms and polysubstance use. SEM refers to *‘a set of equations with accompanying assumptions of the analyzed system, in which the parameters are determined on the basis of statistical observation’* (Tarka, 2018). SEM provides a robust statistical framework for investigating mediation effects (Brown & Little, 2015). Indeed, SEM can account for measurement error, whereas standard mediation analysis does not (Brown & Little, 2015). In SEM, over- and underestimation of mediation effects are reduced by the simultaneous estimation of all the parameters (Hoyle & Smith, 1994). SEM allows for the determination of latent variables, enhancing model identification and interpretation by focusing on underlying constructs rather than individual indicators (Kline, 2011). Latent variables mitigate issues related to multicollinearity among observed variables by capturing their shared variance (Kline, 2011). Finally, SEM allows for the evaluation of multiple mediation effects in a single analysis. Some other studies in mental health have previously used the SEM approach to test mediation (Liu et al., 2022; Sha & Dong, 2021; Tong et al., 2016; Tzang, Chang, & Chang, 2022; Tzang, Chang, & Chang, 2021).

In SEM, latent variables are also referred to as factors. Variables that predict others are called exogenous variables, and variables that are predicted are called endogenous variables. Observed variables predicted by factors are referred to as indicators, and the coefficients of these relationships are known as factor loadings. We followed several steps to perform SEM: specification of the model, identification of the model, fit evaluation of the model, modification of the model, determining the final model, and reporting the results. Estimations have been presented both in raw form with their confidence intervals and standardized. Robust standard errors for the estimations were computed using bootstrapping with 10,000 simulations. Estimations were tested using the Satorra-Bentler test (Satorra & Bentler, 2001).

Data included numerical and categorical variables. We tested the multivariate normality of numerical variables using Mardia’s multivariate skewness and kurtosis (Mardia, 1970). The estimator was diagonally weighted least squares (DWLS) and used a polychoric correlation matrix (Christoffersson, 1977). DWLS makes no specific assumptions about distributions and is less biased and more accurate than other estimators (Li, 2016). DWLS was adapted for categorical data and deviation from multivariate normality. The optimizer was nlminb (Gay, 1990). Estimations have been presented both in raw form with their confidence intervals and standardized. Robust standard errors for the estimations were computed using bootstrapping with 10,000 simulations. Bootstrapping is a resampling method commonly used in SEM (Preacher & Hayes, 2008). Bootstrapping is independent of the distribution of the variable and, thus, is robust to deviations from normality, including multivariate normality.

The adequacy of the model to the data was tested using a conventional chi-square test and various goodness-of-fit indices: standardized root mean square residual (SRMR), root mean square error of approximation (RMSEA), confirmatory fit index (CFI), and Tucker-Lewis index (TLI) (Gana & Broc, 2019). The use of goodness-of-fit indices was necessary because tests for model adequacy are sensitive to large sample sizes (Gana & Broc, 2019). The Satorra-Bentler test assumes violations of assumptions such as multivariate normality and homoscedasticity. The different goodness-of-fit indices are complementary due to their distinct natures. SRMR is an absolute fit index based on a comparison between the observed variance-covariance matrix and the theoretical variance-covariance matrix. RMSEA is a parsimonious fit index that takes into account the parsimony of the theoretical model. CFI and TLI are incremental fit indices based on a comparison of a specified model against a more restrictive nested model, namely the null model. An excellent fit is obtained when SRMR ≤ 0.08, RMSEA ≤ 0.06, and CFI and TLI ≥ 0.95; and an adequate fit is obtained when SRMR and RMSEA ≤ 0.08, and CFI and TLI ≥ 0.90 (Gana & Broc, 2019; Kyndt & Onghena, 2014).

To select adjustment variables, we used a multivariate outcomes and multivariable predictors model. The ASRS total score at T1 and the square root of the number of substance use at T3 were the outcomes, and the adjusting variables were the predictors. Adjusting variables were introduced sequentially in the model. First, we added age at T1, sex, and the number of substance uses at T1. Then, we considered all variables significantly associated with either ASRS total score at T1 or the number of substance uses at T3. To select adjustment variables, we used a bidirectional stepwise selection, keeping variables significantly associated with the two outcomes. Age at T1, sex, and the number of substance uses at T1 were forced into the model. We took into account collinearity using a least absolute shrinkage and selection operator (LASSO) version of the model to remove collinear adjusting variables. Numerical adjusting variables were standardized. Selected adjusting variables were integrated as predictors for both ADHD factors and polysubstance use factors.

We calculated the power for SEM using the method of MacCallum et al. (1996) (MacCallum, Browne, & Sugawara, 1996) with a null hypothesis of RMSEA equal to 0.05.

## 1.5 Multiple groups structural equation modeling, detailed method

Finally, we assessed the stability of the results based on sex. We used a multiple groups SEM (Jöreskog, 1971; Sörbom, 1974) with sex as the grouping variable. This allowed us to study the influence of being female or male on the mediation. First, we imposed a similar structure across the two groups (configural invariance) and then we tested the adequacy of the model. Then, we used a likelihood ratio test, the delta-RMSEA (difference between RMSEA), and the delta-TLI (difference between TLI) to assess various levels of invariance: weak invariance (where factor loadings are constrained to be equal across groups), strong invariance (where both factor loadings and intercepts are constrained), strict invariance (where factor loadings, intercepts, and residual variances are constrained), and structural invariance (where factor loadings, intercepts, residual variances, residual covariances, latent variable variances, and latent variable covariances are constrained to be equal across groups, while regression coefficients are freely estimated). The estimator and optimizer used were the same as those for the main model.

## 1.6 General informations on statistics

First, the significance level was 5%. Statistical tests were two-sided. Estimates were rounded to two decimal places, except p-values, which were rounded to three decimal places. We used R version 4.3.0 (R Core Team, 2023) for basic statistics, the lavaan package version 0.6-18 (Rosseel, 2012) for SEM, and some other packages for various purposes (Jorgensen, Pornprasertmanit, Schoemann, & Rosseel, n.d.; Lishinski, n.d.). Draw.io was used for the flowchart and the SEM representations (“Draw.io,” n.d.).

# 2. Supplementary results

## 2.1 Description of the sample

We see on table S2 the full description of the sample for imputed data.

Tobacco use rate was 16.12% (270) and Cannabis use rate was 26.39% (442). A particular feature of our study sample is that it consisted of students. About 80% of participants were women. The higher rate of cannabis use in comparison of tobacco use could be explained by the fact that female students tended to smoke less tobacco than working men, while students are more likely to experiment with cannabis.

### Table S2: Full description of the sample (n = 1675)

| **Total sample** | **% (n) or mean (sd)** |
| --- | --- |
| ***Student variables at T1:*** |  |
| Sex: female | 80.3 (1345) |
| Age (years) | 20.27 (2.15) |
| Academic level: first three years | 72.54 (1215) |
| Secondary education degree type: not technical | 95.76 (1604) |
| Deprived students' Social support | 40.18 (673) |
| Job activity | 39.58 (663) |
| ***Family variables at T1:*** |  |
| Number of siblings | 1.67 (1.08) |
| Parental academic level: postgraduate | 58.39 (978) |
| Parental separation | 28.12 (471) |
| Parental support during childhood: a lot | 76.18 (1276) |
| ***Psychiatric conditions at T1:*** |  |
| Suicide attempt history | 7.16 (120) |
| Depression history | 9.79 (164) |
| Anxiety disorder history | 13.13 (220) |
| Eating disorder history | 3.64 (61) |
| Obsessive compulsive disorder history | 1.73 (29) |
| ***Scales:*** |  |
| ASRS at T1 | 10.66 (3.99) |
| ASRS attention deficit at T1 | 6.48 (3.16) |
| ASRS hyperactivity at T1 | 4.19 (1.91) |
| PHQ9 at T2 | 6.92 (5.33) |
| STAI-Y2 at T2 | 47.11 (10.57) |
| ***Substance uses at T1:*** |  |
| Substance use | 97.07 (1626) |
| Number of substance use | 2.27 (1.33) |
| Alcohol use | 92 (1541) |
| Tobacco use | 20.3 (340) |
| Cannabis use | 40.24 (674) |
| Cocaine use | 3.28 (55) |
| Ecstasy use | 5.97 (100) |
| Amphetamines use | 1.55 (26) |
| Magic mushrooms use | 38.09 (638) |
| Other drugs use | 25.13 (421) |
| ***Substance uses at T3:*** |  |
| Substance use | 93.49 (1566) |
| Number of substance use | 1.52 (1.04) |
| Alcohol use | 92.96 (1557) |
| Tobacco use | 16.12 (270) |
| Cannabis use | 26.39 (442) |
| Cocaine use | 3.82 (64) |
| Ecstasy use | 4.96 (83) |
| Amphetamines use | 0.72 (12) |
| Magic mushrooms use | 2.03 (34) |
| Other drugs use | 4.84 (81) |
| n: count; %: percentage; m: mean; sd: standard deviation; T1: inclusion; T2: first follow-up; T3: second follow-up; ASRS: Adult ADHD Self-Report Scale; PHQ9: Patient Health Questionnaire - 9; STAI-Y2: State Trait Anxiety Inventory - form Y trait part | |

## 2.2 Individual effects

The table S3 presents the individual effects.

### Table S3: Effects of the mediation analysis in univariate univariable regression models

|  | **Estimate** | **Standard error** | **Exp(estimate)** | **p** |
| --- | --- | --- | --- | --- |
| ***Linear models*** |  |  |  |  |
| ASRS at T1 -> STAI-Y trait at T2 | 0.93 | 0.06 | - | < 0.001 |
| ASRS at T1 -> PHQ-9 at T2 | 0.45 | 0.03 | - | < 0.001 |
| ***Generalized linear models with poisson link*** |  |  |  |  |
| ASRS at T1 -> Number of substance use at T3 | 0.02 | 0 | 1.02 | < 0.001 |
| STAI-Y trait at T2 -> Number of subtance use at T3 | 0 | 0 | 1 | 0.487 |
| PHQ-9 at T2 -> Number of substance use at T3 | 0 | 0 | 1 | 0.976 |
| p: degree of significantly | | | | |

## 2.3 Multinormality

We tested the multinormality of ASRS’ items using using Mardia’s multivariate skewness and kurtosis (Mardia, 1970). The multinormality was not respected with degree of significance < 0.001 for skewness and < 0.001 for kurtosis.

## 2.4 Structural equation modeling, first model

The figure S2 presents the first SEM.

### Figure S2: First SEM


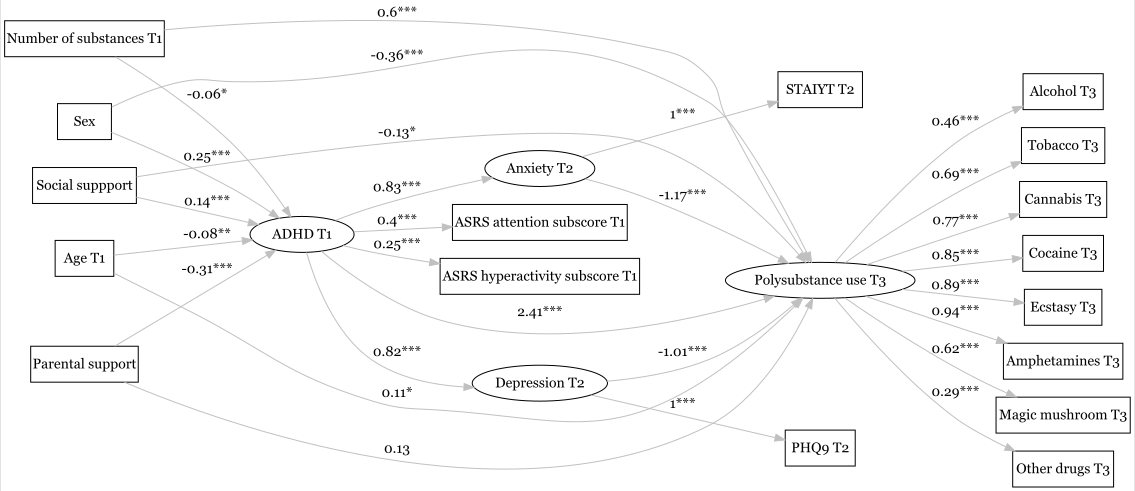

The figure depicts the structural equation model. Ellipses represent factors, rectangles represent observed variables, and single arrows indicate regressions. Starts denote statistical significance (*: <0.05; **: <0.01; ***: <0.001). The estimations are standardized.

The table S4 shows estimations of the factors loadings, intercepts, slopes, thresholds, and error variances for the first model.

### Table S4: Detailed parameters of the first SEM

|  | **Raw Coefficient (95%CI)** | **Standardized Coefficient** |
| --- | --- | --- |
| ***Factor loadings:*** |  |  |
| ADHD -> ASRS attention deficit | 1.01 (0.86 - 1.16) | 0.4*** |
| ADHD -> ASRS hyperactivity | 0.38 (0.28 - 0.47) | 0.25*** |
| Polysubstance use -> Alcohol | 0.54 (0.28 - 0.69) | 0.46*** |
| Polysubstance use -> Tobacco | 0.8 (0.7 - 0.91) | 0.69*** |
| Polysubstance use -> Cannabis | 0.91 (0.81 - 1.01) | 0.77*** |
| Polysubstance use -> Cocaine | 1 | 0.85 |
| Polysubstance use -> Ecstasy | 1.05 (0.96 - 1.13) | 0.89*** |
| Polysubstance use -> Amphetamines | 1.11 (0.99 - 1.26) | 0.94*** |
| Polysubstance use -> Magic mushrooms | 0.73 (0.53 - 0.94) | 0.62*** |
| Polysubstance use -> Other drugs | 0.33 (0.17 - 0.48) | 0.29*** |
| Anxiety -> STAI-Y2 | 3.39 (3.11 - 3.6) | 1*** |
| Depression -> PHQ9 | 2.35 (2.17 - 2.5) | 1*** |
| ***Effects:*** |  |  |
| Direct effect  (ADHD -> Polysubtance use) | 1.66 (1.04 - 2.48) | 2.41*** |
| Indirect effect - Anxiety  (ADHD -> Anxiety -> Polysubtance use) | -0.68 (-1.15 - -0.37) | -0.98*** |
| Indirect effect - Depression  (ADHD -> Depression -> Polysubtance use) | -0.57 (-0.94 - -0.32) | -0.83*** |
| Total effect  (ADHD => Polysubtance use) | 0.41 (0.27 - 0.59) | 0.6*** |
| ***Regressions:*** |  |  |
| ADHD -> Anxiety | 2.08 (1.86 - 2.31) | 0.83*** |
| ADHD -> Depression | 1.49 (1.33 - 1.65) | 0.82*** |
| Anxiety -> Polysubstance use | -0.33 (-0.52 - -0.19) | -1.17*** |
| Depression -> Polysubstance use | -0.39 (-0.6 - -0.22) | -1.01*** |
| Age -> ADHD | -0.1 (-0.17 - -0.04) | -0.08** |
| Sex -> ADHD | 0.31 (0.21 - 0.43) | 0.25*** |
| Number of substance -> ADHD | -0.08 (-0.15 - -0.01) | -0.06* |
| Social support -> ADHD | 0.17 (0.09 - 0.26) | 0.14*** |
| Parental support during childhood -> ADHD | -0.38 (-0.48 - -0.29) | -0.31*** |
| Age -> Polysubtance use | 0.09 (0.03 - 0.17) | 0.11* |
| Sex -> Polysubtance use | -0.31 (-0.46 - -0.18) | -0.36*** |
| Number of substance -> Polysubtance use | 0.52 (0.46 - 0.6) | 0.6*** |
| Social support -> Polysubtance use | -0.11 (-0.22 - -0.02) | -0.13* |
| Parental support during childhood -> Polysubtance use | 0.11 (0.01 - 0.25) | 0.13 |
| ***Intercepts:*** |  |  |
| ASRS attention deficit | 6.48 (6.32 - 6.63) | 6.48 (6.32 - 6.63) |
| ASRS hyperactivity | 4.19 (4.09 - 4.28) | 4.19 (4.09 - 4.28) |
| STAI-Y2 | 47.11 (46.6 - 47.6) | 47.11 (46.6 - 47.6) |
| PHQ9 | 6.92 (6.67 - 7.17) | 6.92 (6.67 - 7.17) |
| Age | 0 (-0.05 - 0.05) | 0 (-0.05 - 0.05) |
| Number of substances | 0 (-0.05 - 0.05) | 0 (-0.05 - 0.05) |
| ***Thresholds:*** |  |  |
| Alcohol | -1.47 (-1.57 - -1.39) | -1.47 (-1.57 - -1.39) |
| Tobacco | 0.99 (0.92 - 1.06) | 0.99 (0.92 - 1.06) |
| Cannabis | 0.63 (0.57 - 0.69) | 0.63 (0.57 - 0.69) |
| Cocaine | 1.77 (1.67 - 1.89) | 1.77 (1.67 - 1.89) |
| Ecstasy | 1.65 (1.56 - 1.76) | 1.65 (1.56 - 1.76) |
| Amphetamines | 2.45 (2.28 - 2.7) | 2.45 (2.28 - 2.7) |
| Magic mushrooms | 2.05 (1.92 - 2.2) | 2.05 (1.92 - 2.2) |
| Other drugs | 1.66 (1.57 - 1.77) | 1.66 (1.57 - 1.77) |
| Sex | -0.85 (-0.92 - -0.78) | -0.85 (-0.92 - -0.78) |
| Social support | 0.25 (0.19 - 0.31) | 0.25 (0.19 - 0.31) |
| Parental support during childhood | -0.71 (-0.78 - -0.65) | -0.71 (-0.78 - -0.65) |
| ***Error variances:*** |  |  |
| ASRS attention deficit | 8.36 (7.73 - 8.97) | 0.84*** |
| ASRS hyperactivity | 3.44 (3.22 - 3.66) | 0.94*** |
| STAI-Y2 | 0 (0 - 0) | 0 |
| PHQ9 | 0 (0 - 0) | 0 |
| Age | 1 (0.92 - 1.08) | 1*** |
| Number of substances | 1 (0.92 - 1.08) | 1*** |
| Alcohol | 0.79 (0.79 - 0.79) | 0.79 |
| Tobacco | 0.54 (0.54 - 0.54) | 0.53 |
| Cannabis | 0.41 (0.41 - 0.41) | 0.4 |
| Cocaine | 0.29 (0.29 - 0.29) | 0.28 |
| Ecstasy | 0.21 (0.21 - 0.21) | 0.2 |
| Amphetamines | 0.13 (0.13 - 0.13) | 0.12 |
| Magic mushrooms | 0.62 (0.62 - 0.62) | 0.61 |
| Other drugs | 0.92 (0.92 - 0.92) | 0.92 |
| Sex | 1 | 1 |
| Social support | 1 | 1 |
| Parental support during childhood | 1 | 1 |
| ADHD | 1.28 (1.15 - 1.43) | 0.81*** |
| Polysubstance use | -0.3 (-1.29 - 0.18) | -0.4 |
| Anxiety | 2.95 (2.7 - 3.2) | 0.3*** |
| Depression | 1.66 (1.52 - 1.8) | 0.32*** |
| CI: 10,000 iterations bootstrapped confident interval; *: p < 0.05; **: p < 0.01; ***: p < 0.001; p: degree of significantly from Satorra - Bentler tests; =>: direct + indirect | | |

## 2.5 Structural equation modeling, final model

The table S5 presents all the estimations for the final model. We found a power of 99% for the model.

### Table S5: Detailed parameters of the final SEM

|  | **Raw Coefficient (95%CI)** | **Standardized Coefficient** |
| --- | --- | --- |
| ***Factor loadings:*** |  |  |
| ADHD -> ASRS attention deficit | 1.06 (0.89 - 1.21) | 0.4*** |
| ADHD -> ASRS hyperactivity | 0.39 (0.29 - 0.49) | 0.24*** |
| Polysubstance use -> Alcohol | 0.64 (0.45 - 0.87) | 0.12*** |
| Polysubstance use -> Tobacco | 1 (0.83 - 1.22) | 0.17*** |
| Polysubstance use -> Cannabis | 1.02 (0.85 - 1.25) | 0.17*** |
| Polysubstance use -> Cocaine | 1 | 0.17 |
| Polysubstance use -> Ecstasy | 0.95 (0.78 - 1.13) | 0.16*** |
| Polysubstance use -> Amphetamines | 0.92 (0.47 - 1.34) | 0.16*** |
| Polysubstance use -> Magic mushrooms | 0.6 (0.34 - 0.87) | 0.11*** |
| Polysubstance use -> Other drugs | 0.15 (-0.05 - 0.36) | 0.03 |
| Anxiety -> STAI-Y2 | 3.56 (3.22 - 3.83) | 1*** |
| Depression -> PHQ9 | 2.34 (2.18 - 2.47) | 1*** |
| ***Effects:*** |  |  |
| Direct effect  (ADHD -> Polysubtance use) | 1.68 (1 - 2.57) | 10.23*** |
| Indirect effect - Anxiety  (ADHD -> Anxiety -> Polysubtance use) | -0.77 (-1.34 - -0.41) | -4.71** |
| Indirect effect - Depression  (ADHD -> Depression -> Polysubtance use) | -0.52 (-0.84 - -0.28) | -3.13*** |
| Total effect  (ADHD => Polysubtance use) | 0.39 (0.24 - 0.57) | 2.39*** |
| ***Regressions:*** |  |  |
| ADHD -> Anxiety | 2.13 (1.91 - 2.39) | 0.85*** |
| ADHD -> Depression | 1.56 (1.38 - 1.73) | 0.81*** |
| Anxiety -> Polysubstance use | -0.36 (-0.59 - -0.2) | -5.52*** |
| Depression -> Polysubstance use | -0.33 (-0.53 - -0.18) | -3.87*** |
| Sex -> ADHD | 0.3 (0.2 - 0.4) | 0.25*** |
| Number of substance -> ADHD | -0.09 (-0.16 - -0.02) | -0.07* |
| Parental support during childhood -> ADHD | -0.36 (-0.45 - -0.28) | -0.3*** |
| Sex -> Polysubtance use | -0.27 (-0.43 - -0.15) | -1.4*** |
| Number of substance -> Polysubtance use | 0.56 (0.46 - 0.66) | 2.87*** |
| Parental support during childhood -> Polysubtance use | 0.1 (0 - 0.22) | 0.51 |
| ***Intercepts:*** |  |  |
| ASRS attention deficit | 6.48 (6.33 - 6.63) | 2.05*** |
| ASRS hyperactivity | 4.19 (4.09 - 4.28) | 2.19*** |
| STAI-Y2 | 47.11 (46.61 - 47.61) | 4.46*** |
| PHQ9 | 6.92 (6.67 - 7.17) | 1.3*** |
| Number of substances | 0 | 0 |
| ***Thresholds:*** |  |  |
| Alcohol | -1.47 (-1.57 - -1.39) | -1.39*** |
| Tobacco | 0.99 (0.92 - 1.06) | 0.87*** |
| Cannabis | 0.63 (0.57 - 0.7) | 0.55*** |
| Cocaine | 1.77 (1.67 - 1.89) | 1.55*** |
| Ecstasy | 1.65 (1.55 - 1.76) | 1.46*** |
| Amphetamines | 2.45 (2.28 - 2.69) | 2.19*** |
| Magic mushrooms | 2.05 (1.92 - 2.2) | 1.94*** |
| Other drugs | 1.66 (1.57 - 1.77) | 1.66*** |
| Sex | -0.85 (-0.92 - -0.78) | -0.85*** |
| Parental support during childhood | -0.71 (-0.78 - -0.65) | -0.71*** |
| ***Error variances:*** |  |  |
| ASRS attention deficit | 8.4 (7.77 - 9.01) | 0.84*** |
| ASRS hyperactivity | 3.45 (3.22 - 3.66) | 0.94*** |
| STAI-Y2 | 0 | 0 |
| PHQ9 | 0 | 0 |
| Number of substances | 1 | 1 |
| Alcohol | 1.11 (1.11 - 1.11) | 0.99 |
| Tobacco | 1.26 (1.26 - 1.26) | 0.97 |
| Cannabis | 1.27 (1.27 - 1.27) | 0.97 |
| Cocaine | 1.26 (1.26 - 1.26) | 0.97 |
| Ecstasy | 1.24 (1.24 - 1.24) | 0.97 |
| Amphetamines | 1.22 (1.22 - 1.22) | 0.97 |
| Magic mushrooms | 1.1 (1.1 - 1.1) | 0.99 |
| Other drugs | 1.01 (1.01 - 1.01) | 1 |
| Sex | 1 | 1 |
| Parental support during childhood | 1 | 1 |
| ADHD | 1.18 (1.06 - 1.33) | 0.84*** |
| Polysubstance use | -0.95 (-1.92 - -0.49) | -25.15** |
| Anxiety | 2.39 (2.16 - 2.6) | 0.27*** |
| Depression | 1.77 (1.63 - 1.91) | 0.34*** |
| ***Covariances:*** |  |  |
| Alcohol - Tobacco | 0.35 (0.22 - 0.54) | 0.3*** |
| Alcohol - Cannabis | 0.57 (0.44 - 0.7) | 0.48*** |
| Alcohol - Cocaine | 0.25 (0.05 - 0.37) | 0.22** |
| Alcohol - Ecstasy | 0.13 (-0.05 - 0.36) | 0.11 |
| Alcohol - Amphetamines | -0.21 (-0.46 - 0.05) | -0.18 |
| Alcohol - Magic_mushroom | -0.07 (-0.27 - 0.21) | -0.06 |
| Alcohol - Other_drugs | 0.21 (0.02 - 0.41) | 0.2* |
| Tobacco - Cannabis | 0.57 (0.47 - 0.67) | 0.45*** |
| Tobacco - Cocaine | 0.45 (0.33 - 0.57) | 0.36*** |
| Tobacco - Ecstasy | 0.48 (0.36 - 0.58) | 0.38*** |
| Tobacco - Amphetamines | 0.39 (0.13 - 0.61) | 0.31** |
| Tobacco - Magic_mushroom | 0.36 (0.19 - 0.5) | 0.3*** |
| Tobacco - Other_drugs | 0.1 (-0.06 - 0.23) | 0.09 |
| Cannabis - Cocaine | 0.57 (0.46 - 0.68) | 0.45*** |
| Cannabis - Ecstasy | 0.59 (0.49 - 0.69) | 0.47*** |
| Cannabis - Amphetamines | 0.26 (0.01 - 0.48) | 0.21* |
| Cannabis - Magic_mushroom | 0.38 (0.23 - 0.52) | 0.32*** |
| Cannabis - Other_drugs | 0.32 (0.2 - 0.43) | 0.28*** |
| Cocaine - Ecstasy | 0.81 (0.7 - 0.9) | 0.65*** |
| Cocaine - Amphetamines | 0.81 (0.64 - 0.91) | 0.65*** |
| Cocaine - Magic_mushroom | 0.55 (0.36 - 0.68) | 0.47*** |
| Cocaine - Other_drugs | 0.28 (0.06 - 0.44) | 0.25** |
| Ecstasy - Amphetamines | 0.87 (0.74 - 0.94) | 0.71*** |
| Ecstasy - Magic_mushroom | 0.59 (0.41 - 0.71) | 0.5*** |
| Ecstasy - Other_drugs | 0.31 (0.11 - 0.45) | 0.28*** |
| Amphetamines - Magic_mushroom | 0.77 (0.53 - 0.91) | 0.66*** |
| Amphetamines - Other_drugs | 0.26 (-0.04 - 0.51) | 0.23 |
| Magic_mushroom - Other_drugs | 0.26 (-0.05 - 0.45) | 0.25* |
| CI: 10,000 iterations bootstrapped confident interval; *: p < 0.05; **: p < 0.01; ***: p < 0.001; p: degree of significantly from Satorra - Bentler tests; =>: direct + indirect | | |

## 2.6 Structural equation modeling, multiple groups

The table S6 presents all parameters for the multiple groups SEM grouped by sex.

### Table S6: Detailed parameters of multiple groups structured equation modeling

|  | **Female group** | |  | **Male group** | |
| --- | --- | --- | --- | --- | --- |
|  | **Raw Coefficient (95%CI)** | **Standardized Coefficient** |  | **Raw Coefficient (95%CI)** | **Standardized Coefficient** |
| ***Factor loadings:*** |  |  |  |  |  |
| ADHD -> ASRS attention deficit | 1.1 (0.91 - 1.28) | 0.41*** |  | 1.13 (0.62 - 1.47) | 0.48*** |
| ADHD -> ASRS hyperactivity | 0.43 (0.32 - 0.55) | 0.26*** |  | 0.3 (0.11 - 0.52) | 0.22** |
| Polysubstance use -> Alcohol | 0.61 (0.37 - 0.95) | 0.39*** |  | 0.93 (0.41 - 1.54) | 0.36** |
| Polysubstance use -> Tobacco | 1.12 (0.87 - 1.56) | 0.65*** |  | 0.96 (0.71 - 1.34) | 0.37*** |
| Polysubstance use -> Cannabis | 1.1 (0.86 - 1.52) | 0.64*** |  | 0.98 (0.7 - 1.43) | 0.37*** |
| Polysubstance use -> Cocaine | 1 | 0.59 |  | 1 | 0.38 |
| Polysubstance use -> Ecstasy | 0.92 (0.69 - 1.22) | 0.55*** |  | 0.95 (0.7 - 1.27) | 0.36*** |
| Polysubstance use -> Amphetamines | 1.02 (0.46 - 1.72) | 0.6** |  | 0.92 (-0.17 - 2.39) | 0.36 |
| Polysubstance use -> Magic mushrooms | 0.44 (0.05 - 0.81) | 0.28* |  | 0.77 (0.34 - 1.22) | 0.31*** |
| Polysubstance use -> Other drugs | 0.18 (-0.08 - 0.47) | 0.12 |  | -0.12 (-0.62 - 0.29) | -0.05 |
| Anxiety -> STAI-Y2 | 3.72 (3.45 - 3.96) | 1*** |  | 3.18 (1.56 - 3.93) | 1 |
| Depression -> PHQ9 | 2.42 (2.22 - 2.6) | 1*** |  | 2.4 (2.08 - 2.66) | 1 |
| ***Effects:*** |  |  |  |  |  |
| Direct effect  (ADHD -> Polysubtance use) | 1.02 (0.44 - 1.68) | 1.82* |  | 1.96 (0.68 - 8.76) | 6.28 |
| Indirect effect - Anxiety  (ADHD -> Anxiety -> Polysubtance use) | -0.39 (-0.7 - -0.16) | -0.69 |  | -1.19 (-7.41 - -0.27) | -3.81 |
| Indirect effect - Depression  (ADHD -> Depression -> Polysubtance use) | -0.37 (-0.69 - -0.11) | -0.65 |  | -0.47 (-1.09 - -0.16) | -1.49 |
| Total effect  (ADHD => Polysubtance use) | 0.27 (0.13 - 0.42) | 0.48* |  | 0.3 (0.08 - 0.64) | 0.97 |
| ***Regressions:*** |  |  |  |  |  |
| ADHD -> Anxiety | 1.91 (1.69 - 2.16) | 0.81*** |  | 2.15 (1.64 - 3.35) | 0.9 |
| ADHD -> Depression | 1.55 (1.36 - 1.77) | 0.83*** |  | 1.13 (0.7 - 1.44) | 0.75*** |
| Anxiety -> Polysubstance use | -0.2 (-0.36 - -0.08) | -0.85** |  | -0.55 (-2.41 - -0.14) | -4.25 |
| Depression -> Polysubstance use | -0.24 (-0.44 - -0.07) | -0.79* |  | -0.41 (-0.89 - -0.15) | -2 |
| Number of substance -> ADHD | -0.08 (-0.15 - 0) | -0.07* |  | -0.04 (-0.23 - 0.13) | -0.03 |
| Parental support during childhood -> ADHD | -0.37 (-0.47 - -0.26) | -0.31*** |  | -0.45 (-0.72 - -0.24) | -0.32** |
| Number of substance -> Polysubtance use | 0.5 (0.36 - 0.63) | 0.76*** |  | 0.56 (0.38 - 0.73) | 1.29*** |
| Parental support during childhood -> Polysubtance use | 0.09 (0 - 0.23) | 0.14 |  | 0.01 (-0.23 - 0.32) | 0.03 |
| ***Intercepts:*** |  |  |  |  |  |
| ASRS attention deficit | 6.43 (6.27 - 6.6) | 2.06*** |  | 6.66 (6.31 - 7.02) | 2.04*** |
| ASRS hyperactivity | 4.21 (4.11 - 4.31) | 2.21*** |  | 4.11 (3.9 - 4.32) | 2.09*** |
| STAI-Y2 | 48.15 (47.63 - 48.7) | 4.69*** |  | 42.85 (41.67 - 44) | 4.02*** |
| PHQ9 | 7.27 (6.99 - 7.56) | 1.37*** |  | 5.47 (4.91 - 6.03) | 1.07*** |
| Number of substances | 0 | 0 |  | 0 | 0 |
| ***Thresholds:*** |  |  |  |  |  |
| Alcohol | -1.44 (-1.55 - -1.35) | -1.39*** |  | -1.6 (-1.88 - -1.39) | -1.42*** |
| Tobacco | 0.99 (0.92 - 1.07) | 0.87*** |  | 0.98 (0.83 - 1.15) | 0.86*** |
| Cannabis | 0.68 (0.6 - 0.75) | 0.6*** |  | 0.45 (0.31 - 0.59) | 0.39*** |
| Cocaine | 1.9 (1.77 - 2.05) | 1.71*** |  | 1.43 (1.25 - 1.66) | 1.25*** |
| Ecstasy | 1.78 (1.66 - 1.91) | 1.62*** |  | 1.3 (1.12 - 1.5) | 1.15*** |
| Amphetamines | 2.52 (2.31 - 2.84) | 2.26*** |  | 2.25 (1.97 - 2.74) | 2*** |
| Magic mushrooms | 2.15 (1.99 - 2.37) | 2.11*** |  | 1.76 (1.55 - 2.03) | 1.61*** |
| Other drugs | 1.74 (1.63 - 1.87) | 1.73*** |  | 1.41 (1.23 - 1.63) | 1.41*** |
| Parental support during childhood | -0.73 (-0.8 - -0.65) | -0.73*** |  | -0.65 (-0.81 - -0.52) | -0.65*** |
| ***Error variances:*** |  |  |  |  |  |
| ASRS attention deficit | 8.14 (7.44 - 8.84) | 0.83*** |  | 8.2 (6.71 - 9.64) | 0.77*** |
| ASRS hyperactivity | 3.36 (3.11 - 3.59) | 0.93*** |  | 3.7 (3.15 - 4.2) | 0.95*** |
| STAI-Y2 | 0 | 0 |  | 0 | 0 |
| PHQ9 | 0 | 0 |  | 0 | 0 |
| Number of substances | 1 | 1 |  | 1 | 1 |
| Alcohol | 0.92 (0.92 - 0.92) | 0.85 |  | 1.11 (1.11 - 1.11) | 0.87 |
| Tobacco | 0.75 (0.75 - 0.75) | 0.58 |  | 1.12 (1.12 - 1.12) | 0.86 |
| Cannabis | 0.76 (0.76 - 0.76) | 0.59 |  | 1.12 (1.12 - 1.12) | 0.86 |
| Cocaine | 0.8 (0.8 - 0.8) | 0.65 |  | 1.13 (1.13 - 1.13) | 0.86 |
| Ecstasy | 0.83 (0.83 - 0.83) | 0.7 |  | 1.11 (1.11 - 1.11) | 0.87 |
| Amphetamines | 0.79 (0.79 - 0.79) | 0.64 |  | 1.11 (1.11 - 1.11) | 0.87 |
| Magic mushrooms | 0.96 (0.96 - 0.96) | 0.92 |  | 1.08 (1.08 - 1.08) | 0.91 |
| Other drugs | 0.99 (0.99 - 0.99) | 0.99 |  | 1 | 1 |
| Parental support during childhood | 1 | 1 |  | 1 | 1 |
| ADHD | 1.22 (1.03 - 1.46) | 0.9*** |  | 1.75 (1.32 - 3.98) | 0.89 |
| Polysubstance use | -0.08 (-0.63 - 0.12) | -0.19 |  | -1.3 (-10.45 - -0.32) | -6.82 |
| Anxiety | 2.65 (2.4 - 2.86) | 0.35*** |  | 2.2 (1.27 - 3.05) | 0.2 |
| Depression | 1.54 (1.37 - 1.68) | 0.32*** |  | 1.99 (1.56 - 2.49) | 0.44 |
| ***Covariances:*** |  |  |  |  |  |
| Alcohol - Tobacco | 0.05 (-0.14 - 0.29) | 0.06 |  | 0.22 (-0.01 - 0.38) | 0.2 |
| Alcohol - Cannabis | 0.27 (0.11 - 0.44) | 0.33 |  | 0.4 (0.16 - 0.55) | 0.36 |
| Alcohol - Cocaine | -0.06 (-0.33 - 0.13) | -0.07 |  | 0.04 (-0.26 - 0.21) | 0.04 |
| Alcohol - Ecstasy | -0.1 (-0.34 - 0.15) | -0.12 |  | -0.03 (-0.41 - 0.19) | -0.03 |
| Alcohol - Amphetamines | -0.37 (-0.64 - -0.18) | -0.44* |  | -0.54 (-0.9 - -0.32) | -0.48 |
| Alcohol - Magic_mushroom | -0.17 (-0.4 - 0.11) | -0.18 |  | -0.22 (-0.6 - 0.01) | -0.2 |
| Alcohol - Other_drugs | 0.22 (-0.01 - 0.37) | 0.23 |  | 0.11 (-0.28 - 0.33) | 0.1 |
| Tobacco - Cannabis | 0.06 (-0.09 - 0.21) | 0.08 |  | 0.49 (0.23 - 0.65) | 0.44 |
| Tobacco - Cocaine | -0.12 (-0.27 - 0.04) | -0.15 |  | 0.53 (0.23 - 0.7) | 0.47 |
| Tobacco - Ecstasy | 0.02 (-0.12 - 0.16) | 0.03 |  | 0.46 (0.16 - 0.62) | 0.41 |
| Tobacco - Amphetamines | -0.04 (-0.42 - 0.3) | -0.05 |  | 0.21 (-0.23 - 0.51) | 0.19 |
| Tobacco - Magic_mushroom | 0.02 (-0.2 - 0.22) | 0.02 |  | 0.5 (0.17 - 0.69) | 0.46 |
| Tobacco - Other_drugs | 0.04 (-0.16 - 0.22) | 0.05 |  | 0.08 (-0.26 - 0.3) | 0.08 |
| Cannabis - Cocaine | 0.1 (-0.05 - 0.26) | 0.12 |  | 0.47 (0.16 - 0.66) | 0.42 |
| Cannabis - Ecstasy | 0.2 (0.04 - 0.37) | 0.25 |  | 0.44 (0.16 - 0.62) | 0.39 |
| Cannabis - Amphetamines | -0.14 (-0.43 - 0.07) | -0.18 |  | 0 (-0.37 - 0.28) | 0 |
| Cannabis - Magic_mushroom | 0.22 (0.06 - 0.38) | 0.26* |  | 0.19 (-0.17 - 0.48) | 0.18 |
| Cannabis - Other_drugs | 0.26 (0.11 - 0.39) | 0.3 |  | 0.26 (0.02 - 0.48) | 0.25 |
| Cocaine - Ecstasy | 0.41 (0.27 - 0.55) | 0.51* |  | 0.7 (0.38 - 0.84) | 0.62 |
| Cocaine - Amphetamines | 0.48 (0.21 - 0.71) | 0.6* |  | 0.38 (-0.02 - 0.61) | 0.34 |
| Cocaine - Magic_mushroom | 0.43 (0.16 - 0.63) | 0.49** |  | 0.31 (-0.12 - 0.53) | 0.28 |
| Cocaine - Other_drugs | 0.2 (-0.15 - 0.44) | 0.23 |  | 0.26 (-0.12 - 0.51) | 0.24 |
| Ecstasy - Amphetamines | 0.49 (0.25 - 0.71) | 0.61** |  | 0.62 (0.16 - 0.82) | 0.56 |
| Ecstasy - Magic_mushroom | 0.28 (-0.03 - 0.49) | 0.31* |  | 0.63 (0.31 - 0.81) | 0.57 |
| Ecstasy - Other_drugs | 0.15 (-0.18 - 0.36) | 0.16 |  | 0.41 (0.09 - 0.63) | 0.39* |
| Amphetamines - Magic_mushroom | 0.57 (0.18 - 0.83) | 0.65** |  | 0.7 (0.25 - 0.89) | 0.64 |
| Amphetamines - Other_drugs | -0.01 (-0.17 - 0.22) | -0.01 |  | 0.57 (0.19 - 0.82) | 0.54* |
| Magic_mushroom - Other_drugs | -0.01 (-0.19 - 0.28) | -0.01 |  | 0.46 (0.03 - 0.71) | 0.44* |
| CI: 10,000 iterations bootstrapped confident interval; *: p < 0.05; **: p < 0.01; ***: p < 0.001; p: degree of significantly from Satorra - Bentler tests; =>: direct + indirect | | | | | |

# References

Abdoli, N., Farnia, V., Salemi, S., Davarinejad, O., Ahmadi Jouybari, T., Khanegi, M., … Behrouz, B. (2020). Reliability and Validity of Persian Version of State-Trait Anxiety Inventory Among High School Students. *East Asian Archives of Psychiatry: Official Journal of the Hong Kong College of Psychiatrists = Dong Ya Jing Shen Ke Xue Zhi: Xianggang Jing Shen Ke Yi Xue Yuan Qi Kan*, *30*(2), 44–47. <https://doi.org/10.12809/eaap1870>

Adewuya, A. O., Ola, B. A., & Afolabi, O. O. (2006). Validity of the patient health questionnaire (PHQ-9) as a screening tool for depression amongst Nigerian university students. *Journal of Affective Disorders*, *96*(1-2), 89–93. <https://doi.org/10.1016/j.jad.2006.05.021>

American Psychiatric Association (Ed.). (1998). *Diagnostic and statistical manual of mental disorders: DSM-IV ; includes ICD-9-CM codes effective 1. Oct. 96* (4. ed., 7. print). Washington, DC.

Azur, M. J., Stuart, E. A., Frangakis, C., & Leaf, P. J. (2011). Multiple imputation by chained equations: What is it and how does it work?: Multiple imputation by chained equations. *International Journal of Methods in Psychiatric Research*, *20*(1), 40–49. <https://doi.org/cfbxfz>

Brown, T. A., & Little, T. D. (2015). *Confirmatory factor analysis for applied research* (Second edition). New York London: The Guilford Press.

Caci, H., Bayle, F. J., & Bouchez, J. (2008). Adult ADHD: Translation and factor analysis of the ASRS-1.1. *European Psychiatry*, *23*, S367–S368. <https://doi.org/b9wzwf>

Caci, H., Didier, C., & Wynchank, D. (2023). Validation and bifactor structure of the French Adult ADHD Symptoms Rating Scale v1.1 (ASRS). *L’Encéphale*, S0013700622002743. <https://doi.org/10.1016/j.encep.2022.11.007>

Carballeira, Y., Dumont, P., Borgacci, S., Rentsch, D., de Tonnac, N., Archinard, M., & Andreoli, A. (2007). Criterion validity of the French version of Patient Health Questionnaire (PHQ) in a hospital department of internal medicine. *Psychology and Psychotherapy*, *80*(Pt 1), 69–77. <https://doi.org/10.1348/147608306X103641>

Christoffersson, A. (1977). Two-step weighted least squares factor analysis of dichotomized variables. *Psychometrika*, *42*(3), 433–438. <https://doi.org/10.1007/BF02293660>

Doi, S., Ito, M., Takebayashi, Y., Muramatsu, K., & Horikoshi, M. (2018). Factorial validity and invariance of the Patient Health Questionnaire (PHQ)-9 among clinical and non-clinical populations. *PLoS ONE*, *13*(7). <https://doi.org/10.1371/journal.pone.0199235>

Donham, G. W., & Ludenia, K. (1984). Cross-validation of the State-Trait Anxiety Inventory with an alcoholic population. *Journal of Clinical Psychology*, *40*(2), 629–631. [https://doi.org/10.1002/1097-4679(198403)40:2<629::aid-jclp2270400244>3.0.co;2-a](https://doi.org/10.1002/1097-4679(198403)40:2%3C629::aid-jclp2270400244%3E3.0.co;2-a)

Draw.io. (n.d.). https://www.drawio.com/.

Gana, K., & Broc, G. (2019). *Structural equation modeling with lavaan*. London : Hoboken, NJ: ISTE Ltd ; John Wilery & Sons, Inc.

Gay, D. M. (1990). *Usage Summary for Selected Optimization Routines* (Computing {{Science Technical Report}} No. 153). Murray Hill: AT&T Bell Laboratories.

Gray, S., Woltering, S., Mawjee, K., & Tannock, R. (2014). The Adult ADHD Self-Report Scale (ASRS): Utility in college students with attention-deficit/hyperactivity disorder. *PeerJ*, *2*, e324. <https://doi.org/gfrgdq>

Green, J. G., DeYoung, G., Wogan, M. E., Wolf, E. J., Lane, K. L., & Adler, L. A. (2018). Evidence for the reliability and preliminary validity of the Adult ADHD Self-Report Scale v1.1 (ASRS v1.1) Screener in an adolescent community sample. *International Journal of Methods in Psychiatric Research*, e1751. <https://doi.org/gfrgdw>

Hoyle, R. H., & Smith, G. T. (1994). Formulating clinical research hypotheses as structural equation models: A conceptual overview. *Journal of Consulting and Clinical Psychology*, *62*(3), 429–440. <https://doi.org/10.1037/0022-006X.62.3.429>

Jöreskog, K. G. (1971). Simultaneous factor analysis in several populations. *Psychometrika*, *36*(4), 409–426. <https://doi.org/10.1007/BF02291366>

Jorgensen, T. D., Pornprasertmanit, S., Schoemann, A. M., & Rosseel, Y. (n.d.). semTools: Useful tools for structural equation modeling.

Kessler, R. C., Adler, L., Ames, M., Demler, O., Faraone, S., Hiripi, E., … Walters, E. E. (2005). [The World Health Organization Adult ADHD Self-Report Scale (ASRS): A short screening scale for use in the general population](https://www.ncbi.nlm.nih.gov/pubmed/15841682). *Psychological Medicine*, *35*(2), 245–256.

Kessler, R. C., Adler, L., Gruber, M. J., Sarawate, C. A., Spencer, T., & Van Brunt, D. L. (2007). Validity of the World Health Organization Adult ADHD Self-Report Scale (ASRS) Screener in a representative sample of health plan members. *International Journal of Methods in Psychiatric Research*, *16*(2), 52–65. <https://doi.org/bxwt3x>

Kiatrungrit, K., Putthisri, S., Hongsanguansri, S., Wisajan, P., & Jullagate, S. (2017). Validity and Reliability of Adult ADHD Self-Report Scale Thai Version (ASRS-V1.1 TH). *Shanghai Archives of Psychiatry*, *29*(4), 218–227. <https://doi.org/gfrgdx>

Kline, R. B. (2011). *Principles and practice of structural equation modeling, 3rd ed* (pp. xvi, 427). New York, NY, US: Guilford Press.

Kroenke, K., Spitzer, R. L., & Williams, J. B. (2001). The PHQ-9: Validity of a brief depression severity measure. *Journal of General Internal Medicine*, *16*(9), 606–613. <https://doi.org/10.1046/j.1525-1497.2001.016009606.x>

Kyndt, E., & Onghena, P. (2014). The Integration of Work and Learning: Tackling the Complexity with Structural Equation Modelling. In C. Harteis, A. Rausch, & J. Seifried (Eds.), *Discourses on Professional Learning* (Vol. 9, pp. 255–291). Dordrecht: Springer Netherlands. <https://doi.org/10.1007/978-94-007-7012-6_14>

Li, C.-H. (2016). The performance of ML, DWLS, and ULS estimation with robust corrections in structural equation models with ordinal variables. *Psychological Methods*, *21*(3), 369–387. <https://doi.org/10.1037/met0000093>

Lishinski, A. (n.d.). lavaanPlot: Path Diagrams for ’Lavaan’ Models via ’DiagrammeR’.

Liu, X., Xia, X., Hu, F., Hao, Q., Hou, L., Sun, X., … Dong, B. (2022). The mediation role of sleep quality in the relationship between cognitive decline and depression. *BMC Geriatrics*, *22*(1), 178. <https://doi.org/10.1186/s12877-022-02855-5>

MacCallum, R. C., Browne, M. W., & Sugawara, H. M. (1996). Power analysis and determination of sample size for covariance structure modeling. *Psychological Methods*, *1*(2), 130–149. <https://doi.org/10.1037/1082-989X.1.2.130>

Mardia, K. V. (1970). Measures of multivariate skewness and kurtosis with applications. *Biometrika*, *57*(3), 519–530. <https://doi.org/10.1093/biomet/57.3.519>

McHugh, M. L. (2012). [Interrater reliability: The kappa statistic](https://www.ncbi.nlm.nih.gov/pmc/articles/PMC3900052). *Biochemia Medica*, *22*(3), 276–282.

Patient Health Questionnaire (PHQ) Screeners. (n.d.). https://www.phqscreeners.com/select-screener/.

Preacher, K. J., & Hayes, A. F. (2008). Asymptotic and resampling strategies for assessing and comparing indirect effects in multiple mediator models. *Behavior Research Methods*, *40*(3), 879–891. <https://doi.org/10.3758/BRM.40.3.879>

R Core Team. (2023). R: A Language and Environment for Statistical Computing. Vienna, Austria: R Foundation for Statistical Computing.

Rosseel, Y. (2012). **Lavaan** : An *R* Package for Structural Equation Modeling. *Journal of Statistical Software*, *48*(2). <https://doi.org/10.18637/jss.v048.i02>

Satorra, A., & Bentler, P. M. (2001). A scaled difference chi-square test statistic for moment structure analysis. *Psychometrika*, *66*(4), 507–514. <https://doi.org/10.1007/BF02296192>

Sha, P., & Dong, X. (2021). Research on Adolescents Regarding the Indirect Effect of Depression, Anxiety, and Stress between TikTok Use Disorder and Memory Loss. *International Journal of Environmental Research and Public Health*, *18*(16), 8820. <https://doi.org/10.3390/ijerph18168820>

Silverstein, M. J., Alperin, S., Faraone, S. V., Kessler, R. C., & Adler, L. A. (2018). Test-retest reliability of the adult ADHD Self-Report Scale (ASRS) v1.1 Screener in non-ADHD controls from a primary care physician practice. *Family Practice*, *35*(3), 336–341. <https://doi.org/gdnkfm>

Sörbom, D. (1974). A General Method for Studying Differences in Factor Means and Factor Structure Between Groups. *British Journal of Mathematical and Statistical Psychology*, *27*(2), 229–239. <https://doi.org/10.1111/j.2044-8317.1974.tb00543.x>

Spielberger, C. D., Bruchon-Schweitzer, M., & Paulhan, I. (DL 1993, cop. 1993). *STAI-Y: Inventaire d’anxiété état-trait forme Y*. Paris, France: Éditions du centre de psychologie appliquée, DL 1993.

Spielberger, C., Gorsuch, R., Lushene, R., Vagg, P., & Jacobs, G. (1983). *Manual for the State-Trait Anxiety Inventory (Form Y1 – Y2)*. *Palo Alto, CA: Consulting Psychologists Press;* (Vol. IV).

Sun, Y., Fu, Z., Bo, Q., Mao, Z., Ma, X., & Wang, C. (2020). The reliability and validity of PHQ-9 in patients with major depressive disorder in psychiatric hospital. *BMC Psychiatry*, *20*, 474. <https://doi.org/10.1186/s12888-020-02885-6>

Tarka, P. (2018). An overview of structural equation modeling: Its beginnings, historical development, usefulness and controversies in the social sciences. *Quality & Quantity*, *52*(1), 313–354. <https://doi.org/10.1007/s11135-017-0469-8>

Thomas, C. L., & Cassady, J. C. (2021). Validation of the State Version of the State-Trait Anxiety Inventory in a University Sample. *Sage Open*, *11*(3), 21582440211031900. <https://doi.org/10.1177/21582440211031900>

Tong, L., Shi, H.-J., Zhang, Z., Yuan, Y., Xia, Z.-J., Jiang, X.-X., & Xiong, X. (2016). Mediating effect of anxiety and depression on the relationship between Attention-deficit/hyperactivity disorder symptoms and smoking/drinking. *Scientific Reports*, *6*, 21609. <https://doi.org/10.1038/srep21609>

Tzang, R.-F., Chang, C.-H., & Chang, Y.-C. (2022). Structural Equation Modeling (SEM): Gaming Disorder Leading Untreated Attention-Deficit/Hyperactivity Disorder to Disruptive Mood Dysregulation. *International Journal of Environmental Research and Public Health*, *19*(11), 6648. <https://doi.org/10.3390/ijerph19116648>

Tzang, R.-F., Chang, Y.-C., & Chang, C.-H. (2021). Structural Equation Modeling (SEM): Childhood Aggression and Irritable ADHD Associated with Parental Psychiatric Symptoms. *International Journal of Environmental Research and Public Health*, *18*(19), 10068. <https://doi.org/10.3390/ijerph181910068>

van Buuren, S., & Groothuis-Oudshoorn, K. (2011). mice: Multivariate imputation by chained equations in r. *Journal of Statistical Software*, *45*(3), 1–67.

Van De Glind, G., Van Den Brink, W., Koeter, M. W. J., Carpentier, P.-J., Van Emmerik-van Oortmerssen, K., Kaye, S., … Levin, F. R. (2013). Validity of the Adult ADHD Self-Report Scale (ASRS) as a screener for adult ADHD in treatment seeking substance use disorder patients. *Drug and Alcohol Dependence*, *132*(3), 587–596. <https://doi.org/10.1016/j.drugalcdep.2013.04.010>

Vitasari, P., Wahab, M. N. A., Herawan, T., Othman, A., & Sinnadurai, S. K. (2011). Re-test of State Trait Anxiety Inventory (STAI) among Engineering Students in Malaysia: Reliability and Validity tests. *Procedia - Social and Behavioral Sciences*, *15*, 3843–3848. <https://doi.org/10.1016/j.sbspro.2011.04.383>

Wiglusz, M. S., Landowski, J., & Cubała, W. J. (2019). Psychometric properties and diagnostic utility of the State-Trait Anxiety Inventory in epilepsy with and without comorbid anxiety disorder. *Epilepsy & Behavior: E&B*, *92*, 221–225. <https://doi.org/10.1016/j.yebeh.2019.01.005>
